# Tumour immune contexture and immune evasion in sporadic and Lynch syndrome-associated microsatellite unstable colorectal cancers

**DOI:** 10.1038/s41416-025-03302-z

**Published:** 2026-01-14

**Authors:** Samantha Martin, Hanna Elomaa, Juha P. Väyrynen, Maarit Ahtiainen, Erkki-Ville Wirta, Jan Böhm, Toni T. Seppälä, Ari Ristimäki, Kyösti Tahkola, Anne Mattila, Selja Koskensalo, Laura Renkonen-Sinisalo, Anna Lepistö, Jukka-Pekka Mecklin, Kimmo Palin, Kristiina Rajamäki, Lauri A. Aaltonen

**Affiliations:** 1 Medicum/Department of Medical and Clinical Genetics, University of Helsinki, Helsinki, Finland; 2 Applied Tumor Genomics Research Program, Research Programs Unit, University of Helsinki, Helsinki, Finland; 3 Research Program in Systems Oncology, University of Helsinki, Helsinki, Finland; 4 Translational Medicine Research Unit, University of Oulu, Medical Research Center Oulu, and Oulu University Hospital, Oulu, Finland; 5 Central Finland Biobank, Hospital Nova of Central Finland, Well Being Services County of Central Finland, Jyväskylä, Finland; 6 Department of Gastroenterology and Alimentary Tract Surgery, Tampere University Hospital, Tampere, Finland; 7 Faculty of Medicine and Health Technology, Tampere University and Tays Cancer Center, Tampere University Hospital, Tampere, Finland; 8 Department of Pathology, Hospital Nova of Central Finland, Well Being Services County of Central Finland, Jyväskylä, Finland; 9 Department of Surgery, Helsinki University Central Hospital, Hospital District of Helsinki and Uusimaa, Helsinki, Finland; 10 iCAN Digital Precision Cancer Medicine Flagship, University of Helsinki, Helsinki, Finland; 11 Department of Pathology, HUSLAB, HUS Diagnostic Center, University of Helsinki and Helsinki University Hospital, Helsinki, Finland; 12 Department of Surgery, Wellbeing Services County of Central Finland, Hospital Nova of Central Finland, Jyväskylä, Finland; 13 The HUCH Gastrointestinal Clinic, Helsinki University Central Hospital, Helsinki, Finland; 14 Department of Education and Research, Hospital Nova of Central Finland, Well Being Services County of Central Finland, Jyväskylä, Finland; 15 Department of Sport and Health Sciences, University of Jyväskylä, Jyväskylä, Finland

**Keywords:** Tumour immunology, Colorectal cancer, Cancer genomics

## Abstract

**Background:**

The high mutational burden in microsatellite unstable colorectal cancers (MSI CRCs) results in high immunogenicity, yet response rates to immunotherapy vary, suggesting underlying heterogeneity of the tumour immune landscape. Here, our aims were (1) to characterise the immune cell infiltrate and immune evasion in MSI CRCs, (2) to correlate these with clinical and genomic features, and (3) to compare these between Lynch syndrome (LS) and sporadic MSI CRCs.

**Method:**

Immunohistochemistry was utilised to detect T cell and myeloid cell subsets. Whole-genome and RNA sequencing were utilised to analyse somatic variants, tumour clonality, neoantigen burden, antigen presentation, immune checkpoint expression, and consensus molecular subtypes.

**Results:**

Our results revealed higher immune cell scores in LS tumours, depicting higher T cell infiltration, compared to sporadic tumours. Conversely, sporadic tumours displayed increased infiltration of protumorigenic M2-like macrophages and increased expression of immune checkpoints *PDCD1LG2* and *CD40LG*. Across our MSI CRC cohort, high neoantigen burden was associated with low tumour clonality.

**Conclusions:**

Our findings reveal differences between sporadic MSI and LS tumours in T cell and myeloid immune cell landscapes, and in immune evasion. These differences may contribute to the variable immunotherapy responses among MSI CRC patients and are targetable by emerging therapeutic approaches.

## Introduction

Microsatellite unstable colorectal cancer (MSI CRC) is a hypermutated subgroup of CRC characterised by a defective mismatch repair (MMR) system caused by pathogenic alterations in MMR genes *MLH1*, *MSH2*, *MSH6* or *PMS2*. Approximately 15% of CRCs are MMR-defective, of which approximately 20% are associated with a hereditary cancer syndrome, Lynch Syndrome (LS), while the remaining are sporadic [1]. LS patients inherit a heterozygous germline mutation in an MMR gene, and somatic inactivation of the second allele is required for tumorigenesis. In contrast, bi-allelic hypermethylation of the *MLH1* promoter with consequent downregulation of gene expression is most commonly responsible for MMR impairment in sporadic MSI CRC.

The hypermutated nature of MSI CRC results in an abundance of neoantigens derived from frameshift peptides [2–6]. These are presented on the tumour cell surface as part of HLA class I and class II complexes [7, 8]. Thus, T cells can recognise and target MSI tumour cells, resulting in high immunogenicity and a strong immune response. MSI CRCs are known to have greater T cell infiltration than non-hypermutated microsatellite stable (MSS) CRCs [5, 9–11], and this has been robustly associated with good prognosis and response to immunotherapy in MSI CRCs [5, 12–14]. Consequently, MMR deficiency is often used as a criterion to determine eligibility for immunotherapy in CRC treatment. While dramatic and sustained clinical responses to immunotherapy can be achieved, and among non-metastatic MSI CRCs a complete pathological response is achieved in over 60% and up to 100% in rectal cases [15–19], the response rates among metastatic MSI CRCs range from 30 to 50% implying mechanisms of intrinsic resistance [20].

Multiple mechanisms of immune evasion and escape are observed in established tumours to evade the immune response. These include impaired antigen presentation, expression of immune checkpoint proteins, and production of immunosuppressive factors such as cytokines and chemokines [7, 21]. In MSI CRC, somatic mutations in *B2M* and HLA class I genes are frequently observed, along with up- and downregulation of genes involved in antigen processing and presentation [22, 23]. Furthermore, multiple immune checkpoint molecules are upregulated in MSI compared to MSS CRCs [11].

Innate immune cells of myeloid origin comprise an abundant and functionally diverse population of tumour-associated immune cells. They profoundly shape the immunosuppressive tumour environment, and contribute to poor outcomes and resistance to chemotherapy and immune checkpoint inhibitors [24, 25]. Myeloid cells, such as macrophages and neutrophils, display dynamic phenotypes and can have pro- or anti-tumorigenic effects depending on their functional activation state, i.e. polarisation [24, 26]. In CRC, the polarisation state of tumour-associated macrophages, rather than their overall density, is associated with cancer-specific survival [27]. Higher densities of both intraepithelial and stromal monocytic cells, granulocytic cells, and macrophages are found in MSI compared to MSS CRCs [27, 28]. Myeloid cells may be particularly important in the highly T cell-infiltrated MSI CRCs, as they have the ability to suppress anticancer adaptive immune responses [24].

One aspect potentially contributing to the heterogeneous treatment responses of MSI CRCs to immunotherapy is the differences between LS-associated and sporadic MSI tumours. Given the low prevalence of LS, studies including MSI CRCs without identifying the LS cases are more informative of sporadic MSI CRCs. Neoantigen burden is significantly increased in LS compared to sporadic MSI CRCs [29, 30]. Several studies have found significantly higher T cell infiltration in LS tumours compared to sporadic MSI CRCs [29, 31], while others have found no significant difference [30, 32, 33]. An important qualitative difference is that T cell reactivity against frameshift neoantigens is already detectable in healthy carriers of LS-associated germline mutations [34], suggesting a vaccination-like effect. Little is known about myeloid immune cell infiltration in LS compared to sporadic MSI CRCs.

We recently showed that LS MSI CRCs tend to be more clonal compared to sporadic MSI CRCs based on allelic fraction variance of somatic mutations [35]. We further showed differences between LS and sporadic MSI CRCs in gene expression of immune pathways related to T cell and myeloid immune cell function, and in the estimated proportions of T cell and several myeloid immune cell subsets based on deconvolution analysis of RNA sequencing data [35]. The objective of this investigation was to further elucidate the immunogenic differences between LS and sporadic MSI CRCs. Immunohistochemistry (IHC) assays were performed to reveal immune cell infiltration in the tumours. Conventional IHC and immune cell score calculation were utilised for T cells, whereas myeloid immune cells of the granulocytic and monocytic lineages were quantified using multiplex IHC. The same set of samples was previously whole-genome sequenced, and RNA sequenced [35], allowing comparisons of immune cell infiltration to somatic variant counts, tumour clonality and consensus molecular subtypes [36], and enabling the comprehensive characterisation of immune checkpoint molecule expression, somatic variants in antigen presentation-related molecules, and neoantigen burden [37].

## Materials and methods

### Patient samples

All tissue samples were collected between 1994 and 2017 from CRC patients in Finland, and we have access to detailed clinical information for all patients. The complete sample set consists of 43 MSI CRCs, including 29 sporadic MSI tumour-normal pairs and 14 *MLH1*-defective Lynch Syndrome tumour-normal pairs from 13 patients; the clinical features were summarised previously [35]. The MSI status of the CRCs had been determined previously by a combination of radioactive labelling techniques and fluorescence-based PCR methods as described elsewhere [38]. WGS and RNA-seq analyses were performed with a starting material of fresh-frozen adenocarcinoma tissue, and where possible, corresponding normal colorectal tissue. All 43 tumours were RNA sequenced, and 40 were whole-genome sequenced, as described previously [35]. Conventional IHC stainings of T cells and multiplex IHC stainings of myeloid immune cells were performed on whole sections of formalin-fixed paraffin-embedded (FFPE) tumour tissues. We were able to retrieve FFPE blocks and successfully perform T cell IHC staining for 6 LS and 20 sporadic tumours, and myeloid immune cell IHC staining for 7 LS and 20 sporadic tumours. There was an overlap of 5 LS and 19 sporadic tumours. None of the LS patients are known to be close relatives. Five of the MSI CRCs in this research were rectal tumours, none of which were treated with radiotherapy or chemoradiotherapy prior to surgery. No other neoadjuvant therapy was used as standard of care in Finland during the period of time that these samples were collected.

### Immune cell score

Whole-section slides from FFPE tumours were stained as described [39] with anti-CD3 (LN10, 1:200; Novocastra) and anti-CD8 (SP16, 1:400; Thermo Scientific) antibodies separately to identify total and cytotoxic T cells, respectively. Positively stained cells were analysed using QuPath (v0.2.3) [40] as previously described [32]. The immune cell score was formulated as described [41] following the original method by Galon et al. [42]. Briefly, the score describes the densities (cells/mm²) of total and cytotoxic T cells in the tumour centre and the tumour invasive margin. Cut-off values for cell densities in each location were selected from receiver operating characteristic curves in relation to disease-specific 3-year mortality in an independent patient cohort of 265 tumours, including both MSS and MSI CRCs. The immune cell scores from 223 MSS CRCs from this previous sample set were included in this study. A high density of each T cell subtype in each tumour location contributes one point, giving a maximum immune cell score of 4; low densities of both T cell subtypes in both tumour locations yield the score 0. In the analyses presented, the immune cell scores for the tumours are divided into high (score 3–4) vs low (0–2) groups, representing high and low T cell infiltration, respectively.

### Multiplex immunohistochemistry

Multiplex IHC was used for cyclic detection of target proteins from a single section of a FFPE tumour specimen using the ethanol-soluble chromogen AEC (3-Amino-9-Ethylcarbazole; AEC+ High Sensitivity Substrate Chromogen, Dako, K3469) and Leica Bond-III automated IHC stainer (Leica Biosystems). The set of marker stainings (CD66b, CD14, HLA-DR, CD11b, CD68, CD206, CD16, eosin and CK) was designed for detailed characterisation of different myeloid immune cell populations in the samples. The antibodies and conditions used for each are listed in Supplementary Table 1 in the order used in the consecutive staining cycles; destaining with ethanol was performed between each staining cycle. The stainings were completed using Bond Polymer Refine Detection Kit according to the manufacturer’s instructions (Leica Biosystems, DS9800), replacing chromogen DAB with AEC. Haematoxylin-eosin (H&E) staining was carried out using the Tissue-Tek Prisma Slide Stainer model E2S (Sakura). Coverslipping was performed using Tissue-Tek Glas Automated Glass Coverslipper (Sakura) and VectaMount AQ Aqueous Mounting Medium (Vector Laboratories, H-5501). The stained sections were scanned using the NanoZoomer XR slide scanner (Hamamatsu) at 20× magnification.

After completion of the 8-plex staining panel, the digitised images of multiplex immunohistochemistry slides were processed with QuPath. A total of four 2-mm diameter regions of interest, two from the tumour centre (CT) and two from the invasive margin (IM), were separated into single images for further analyses. Regions that were necrotic or contained minimal amounts of tumour were avoided. The single regions of all staining cycles were combined into a composite pseudo-immunofluorescence image using Fiji/ImageJ.

The pseudo-immunofluorescence images were analysed in QuPath using supervised machine learning-based algorithms [40]. Cells were detected and phenotyped into granulocytes (CD66b + CD14-CK-), monocytic cells (CD14 + CD66b-CK-), tumour cells (CK+CD66b-CD14-), and other cells (CD66b-CD14-CK-). Tissue compartments were categorised into tumour and stroma. Necrotic and empty white spaces were excluded. Further cell classification and analyses were conducted in RStudio and R statistical programming. The mIHC staining and image processing methods have been described in detail previously [43].

Macrophages were defined as CD14 + CD68+ cells. A polarisation index was calculated to predict M1-like and M2-like classifications of the macrophages (HLA-DR - median(HLA-DR) / mad(HLA-DR)) - (CD206 - median(CD206) / mad(CD206)). The top and bottom 30% of macrophages based on their polarisation index were annotated as M1-like and M2-like macrophages, respectively [27].

### Spatial analyses of immune cells

The spatial proximity of the myeloid immune cell subtypes with tumour cells was analysed in the multiplex IHC-stained tumour sections as previously described [43]. Briefly, the spatstat (v2.2–0) package was utilised to calculate the nearest neighbour distances (NNDs) for each immune cell of a specific category to the closest tumour cell. The average NNDs for each immune cell subtype were compared between LS and sporadic MSI tumours. The NND difference between sporadic and LS tumours was calculated as the difference between medians across all cells ((median NND across LS tumours - median NND across sporadic tumours)/median NND across LS tumours).

### RNA sequencing

RNA was extracted from fresh-frozen tissue for all 43 MSI CRCs and, where possible, their paired normal tissue, and sequencing was performed with the Illumina NovaSeq600 platform as previously described [35]. The RNA-seq data was aligned to the GRCh38 reference genome. Differential gene expression analysis was performed comparing LS and sporadic MSI tumours using a DESeq2 model design, which included tumour percentage and scaled RIN as covariates [44]. Unadapted shrinkage of the log2 fold change (LFC) estimates was performed with apeglm, and genes with an adjusted *P*-value < 0.1 and |LFC | > 0.6 were considered differentially expressed [45].

Clustering was performed with DeSeq2 VST-normalised transcript counts [44], and the heatmap was made with the pheatmap R package [46] (v1.0.12).

Consensus molecular subtypes were extracted from the RNA-seq data with the single-sample classifier of CMSclassifier R package v1.0.0 [36] to call the consensus molecular subtype (CMS) for each tumour based on transcriptomic data, expressed here as the nearest CMS (RF.1) predicted by the classifier.

### Whole-genome sequencing

DNA was extracted from fresh-frozen tissue with standard methods from 40 MSI CRCs and matched normal tissue pairs, and paired-end sequencing was performed with the Illumina NovaSeq6000 platform as previously described [35]. The sequences were aligned to the GRCh38 reference genome. A workflow related to the GATK4 best practices (v4.0.4.0) was followed for the pre-processing of the DNA sequence data and subsequent somatic variant calling.

### Somatic variant calling and annotation

Somatic variant annotations were extracted with BasePlayer (v1.0.2) [47] with no additional filters applied. Only somatic variants that were annotated as “PASS” by Mutect2 (v4.0.0.0) were included in the analysis [48]. The cBioPortal tool Oncoprinter was used to generate oncoprint images [49, 50]. Variant effect predictions required as input for the Oncoprinter tool were generated based on the somatic variant annotations from Baseplayer [47]. Neoantigen burden predictions were generated from the somatic variants using the NeoPredPipe pipeline (commit b087fae) [37], using netMHC-pan-4.1 for predicting the HLA class I genotypes [51].

### Statistics

Unless otherwise mentioned, statistical analyses were performed with R (v4.3.1 or v4.0.3) and unadjusted *P*-values are reported. Data was plotted with ggplot2 (v3.5.0). Where possible, scripts are available on Zenodo under the DOI 10.5281/zenodo.15082646. The aspired sample size was 20 per group, providing 80% power for an effect of 1 standard deviation with 5% significance; alas, this sample size was not reached for LS patients due to sample availability.

## Results

### T cell infiltration and immune cell score

**Fig. 1:**
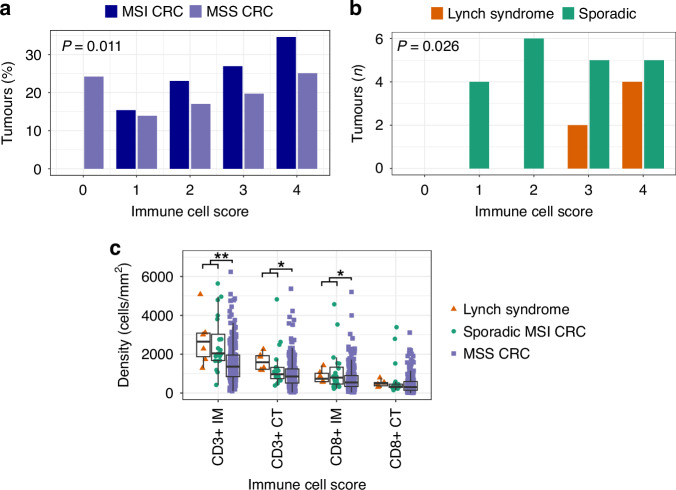
The immune cell scores of 6 LS, 20 sporadic MSI CRCs and 223 MSS CRCs. **a** The proportion of tumours per immune cell score in MSS vs all 26 MSI CRCs. **b** The immune cell scores of LS vs sporadic MSI CRCs. **c** The density of CD3+ and CD8 + T cells/mm^2^ in the tumour centre (CT) and invasive margin (IM). Statistically significant differences are indicated in the figure (**a**, **b** Cochran Armitage test, **c** Mann–Whitney U test, unadjusted *P < 0.05, **P = 2.39 × 10^−^^−^^5^). Other differences are not statistically significant.

T cell infiltration in the tumours was quantified using the immune cell score, demonstrated to be a strong prognostic factor in CRC and evaluated also in clinical settings [42]. The method is based on immunohistochemical stainings of total T cells (CD3+) and cytotoxic T cells (CD8+), followed by analysis of cell densities at the tumour centre and invasive margin. The immune cell score was determined for 6 LS-associated MSI CRCs, 20 sporadic MSI CRCs, and also compared to scores of 223 MSS CRCs that had previously undergone immune cell scoring [39]. As expected, given their hypermutated nature, MSI CRCs showed a statistically significant trend for higher immune cell scores compared to MSS CRCs and displayed no score 0 tumours, reflecting higher T cell infiltration (Fig. 1a; Cochran Armitage test, *P* = 0.011). Among the MSI tumours, all LS tumours had high immune cell scores (3-4) while the sporadic MSI tumours were equally split between high and low (1-2) immune cell scores (Fig. 1b, Supplementary Fig. 1). LS tumours showed a statistically significant trend towards higher immune cell scores (Fig. 1b; Cochran Armitage test, *P* = 0.026). Considering raw T cell densities, the largest increase in density in MSI vs MSS tumours was seen in CD3+ T cells at the invasive margin (895.1 cells/mm^2^ CI_95_[508.2, 1305.5], Mann–Whitney U test, *P* = 2.39 × 10^−^^5^), and for LS vs sporadic MSI tumours in CD3+ T cells at the tumour centre (545.4 cells/mm^2^ CI_95_[−18.3, 1107.8], Mann–Whitney U test *P* = 0.062; Fig. 1c).

### Myeloid immune cell infiltration

**Fig. 2:**
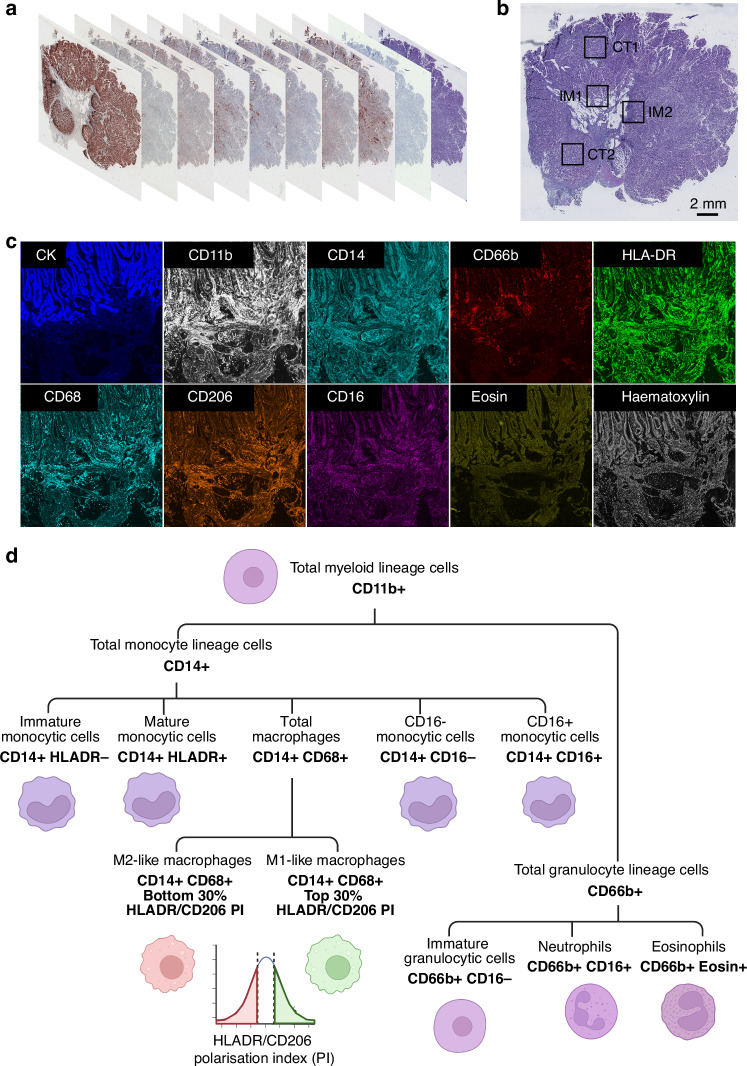
Multiplex immunohistochemistry (mIHC) of myeloid cells. **a** Unprocessed mIHC images from each cycle of marker staining for one tumour. **b** Four 2 × 2 mm regions of interest were selected per tumour, two from each of the tumour centre (CT) and invasive margin (IM). **c** Pseudo-immunofluorescence of markers in one IM region of interest. **d** A schematic of the antibody marker combinations assigned for each myeloid immune cell subset analysed in this study. See “Materials and methods” section for details regarding the calculation of the macrophage polarisation index. Created in BioRender. Martin (2025) https://BioRender.com/y19w074.

To capture the diversity of myeloid immune cell phenotypes in MSI CRC, 7 LS and 20 sporadic MSI tumours were stained using multiplex immunohistochemistry with markers of both granulocytic and monocytic lineages (Supplementary Table 2; Fig. 2). A single tumour section was sequentially stained with each antibody and imaged. In the image analysis, regions of interest were analysed both from the tumour centre (CT) and invasive margin (IM), and immune cells were also stratified according to their location within the tumour epithelium or stroma (Fig. 2).

**Fig. 3:**
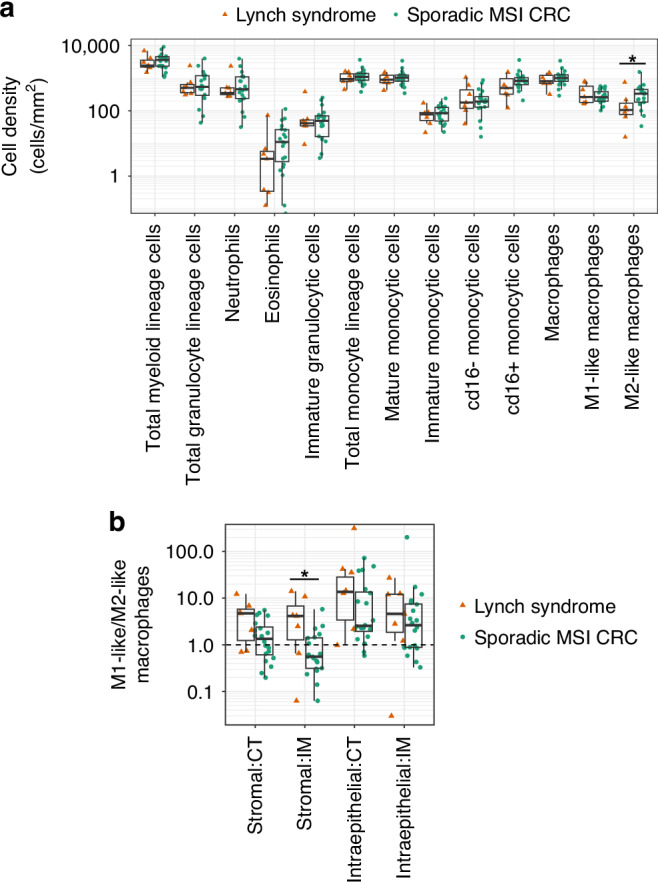
Myeloid immune cell infiltration in 7 LS and 20 sporadic MSI tumours. **a** The myeloid cell densities across all 4 regions of interest analysed from each tumour. The y-axis is on a log scale. *P = 0.048 (Mann–Whitney U test, unadjusted). **b** The ratio of M1-like/M2-like macrophages stratified according to their stromal or intraepithelial location within the tumour centre (CT) and invasive margin (IM). *P = 0.031 (Mann–Whitney U test, unadjusted).

Monocyte lineage cells dominated over granulocyte lineage cells across the CT and IM in both tumour groups, and mature cell subsets dominated over immature cells for both lineages (Fig. 3a, Supplementary Fig. 2). The only statistically significant difference between sporadic MSI and LS tumours was the increased infiltration of M2-like macrophages in sporadic MSI tumours when considering the CT and IM together (2584.5 cells/mm^2^ CI_95_[3, 5438], Mann–Whitney U test *P* = 0.048; Fig. 3a). This cell phenotype has been associated with protumorigenic and immunosuppressive functions [26]. Overall, M1-like macrophages, known to have anti-tumorigenic proinflammatory functions [26], dominated over M2-like macrophages in both tumour groups (Fig. 3b). A striking exception was the IM stroma in sporadic MSI tumours that was dominated by M2-like macrophages and showed a significantly lower M1-like to M2-like macrophage ratio compared to LS tumours (2.8 CI_95_[0.1, 9.3], Mann–Whitney U test, *P* = 0.031; Fig. 3b).

### Spatial interactions of myeloid immune cells and tumour cells

The proximity of myeloid cells to tumour cells could promote tumour-induced changes in their phenotype and function, or enable cell-cell contact for direct antitumour effects (e.g. phagocytosis or cytotoxic activity) [24]. In CRC, myeloid cell proximity to tumour cells independently contributes to their prognostic significance [28]. To compare the spatial immune environment in sporadic MSI and LS tumours, the nearest neighbour distance (NND) was calculated between each identified myeloid immune cell and its nearest tumour cell in the multiplex IHC images. When the measurements were pooled per tumour group, most myeloid immune cells showed significantly shorter median distances to the nearest tumour cell in sporadic MSI compared to LS tumours (Wilcoxon test with Bonferroni correction, adjusted *P* < 0.0001; Supplementary Fig. 3A). However, when comparing the median NNDs per tumour to account for interindividual variation, there were no statistically significant differences between the tumour groups (Supplementary Fig. 3B).

### Correlations between T cell and myeloid immune cell infiltration

Myeloid immune cells can either boost or suppress antitumour T cell responses depending on the myeloid cell subset and differentiation/activation status [24, 25]. However, T cell and myeloid cell densities have rarely been studied in the same tumours. To address their relationship in MSI CRCs, we first compared the myeloid cell densities to the immune cell scores (Supplementary Fig. 4A). When comparing between LS and sporadic MSI CRCs with a high immune cell score, no statistically significant differences were observed for any myeloid cell subset (Mann–Whitney U test). Among sporadic MSI CRCs, tumours with a high immune cell score showed significantly increased infiltration of M1-like macrophages compared to those with a low immune cell score (Mann–Whitney U test, *P* = 2.3 × 10^−^^4^; Supplementary Fig. 4B). The number of total monocyte lineage cells, mature monocytic cells and total macrophages, cell types which encompass M1-like macrophages, were also significantly higher in sporadic tumours with a high immune cell score. Conversely, a significant difference was not seen for M2-like macrophage infiltration, which was elevated in sporadic tumours regardless of their immune cell score (Supplementary Fig. 4B), nor for any of the granulocytic subsets.

The correlation of raw T cell and myeloid cell densities (cells/mm^2^) was tested with a linear regression model with all sporadic and LS MSI CRCs included together. Densities of most monocytic and macrophage subsets positively correlated with CD3+ and CD8+ T cell infiltration, whereas granulocytic subsets showed no correlation (Supplementary Fig. 5).

### Comparison of immune cell infiltration to clinical and genomic features

The immune cell score is a strong predictor of CRC patient survival, even superior to MSI [5, 14]. As expected, MSI CRC patients with high immune cell score tumours showed a trend towards improved CRC-specific survival (Supplementary Fig. 6A). Among the high immune cell score tumours, CRC-specific survival did not significantly differ between sporadic MSI and LS (Supplementary Fig. 6B). The age, gender and distal-proximal tumour location did not correlate with the immune cell scores (Supplementary Fig. 7). The only significant correlations of clinical features with myeloid cell densities were the significantly higher density of immature granulocytic cells and CD16- monocytic cells in male patients and in those who later died from CRC (Supplementary Fig. 7).

**Fig. 4:**
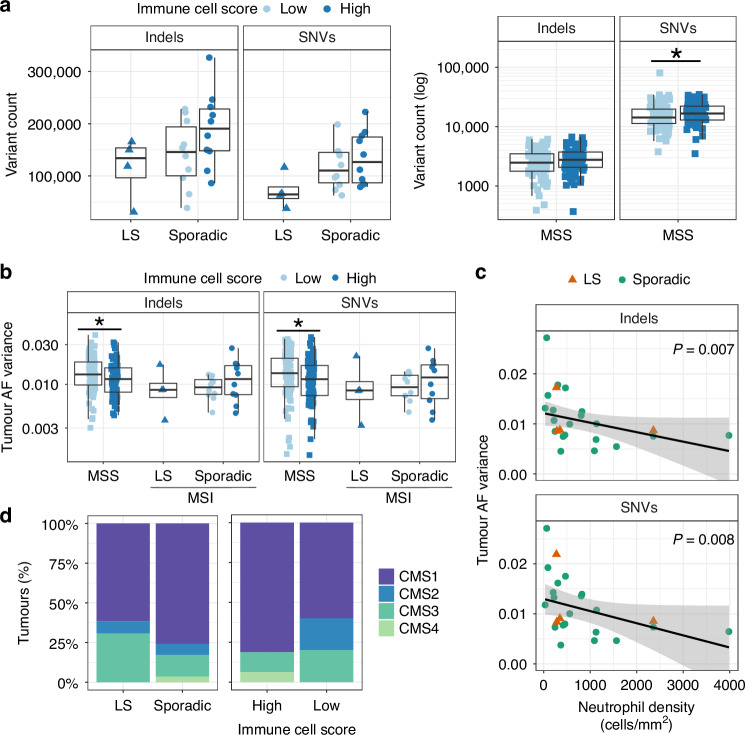
Comparison of immune cell infiltration to genomic features in LS and sporadic MSI CRCs. **a** The total number of somatic SNVs and indels and **b** their allelic fraction (AF) variances in high vs low immune score tumours in 4 LS, 20 sporadic MSI CRCs and 177 MSS CRCs. The number of mutations and AF variance were not significantly different between high and low immune cell score MSI tumours; significant differences between MSS tumours in a Mann–Whitney U test are indicated by an asterisk or *P*-value (*P = 0.04, 0.037 and 0.035 for SNV count, indel AF variance, and SNV AF variances, respectively). **c** The correlation between neutrophil density and AF variance. **d** The distribution of consensus molecular subtypes (CMS) in MSI CRCs by tumour subgroup and by immune cell score.

Tumour mutational burden predicts response to immune checkpoint inhibition in both MSS and MSI CRCs [52, 53], implying an association with neoantigen burden and tumour immune contexture. In our cohort of MSI CRCs, the genome-wide burden of somatic variants was similar in sporadic MSI and LS tumours [35] and not significantly different between high and low immune cell score MSI tumours (Fig. 4a; Supplementary Fig. 8), nor associated with myeloid immune cell densities (Supplementary Fig. 9). The variability in immune cell scores among sporadic MSI tumours was not associated with *BRAF* mutation status (*BRAF* mutants 5/10 and 5/10 in high and low immune cell score tumours, respectively). For our larger cohort of MSS CRCs, significantly higher SNV counts, but not indel counts, were found in high immune cell score tumours compared to low immune cell score tumours (Fig. 4a).

Clonally heterogeneous, i.e., less clonal, tumours have been associated with decreased immunosurveillance [54]. We previously observed that sporadic MSI tumours were less clonal compared to LS tumours in our cohort [35]. We utilised here the same proxy of tumour clonality, allelic fraction (AF) variance of somatic variants, to compare tumour clonality and immune cell infiltration; a high variance reflects low clonality [55]. In agreement with data from other tumour types [54], MSS CRCs with a low immune cell score showed a statistically significantly higher AF variance (Mann–Whitney U test; Fig. 4b), suggesting that MSS tumours with low T cell infiltration were less clonal. This association was lost in MSI CRCs; there was no statistical difference in AF variances of sporadic MSI tumours with high and low immune cell scores (Mann–Whitney U test; Fig. 4b). The densities of total myeloid cells, granulocytic lineage cells, and neutrophils (comprising the majority of granulocytic lineage cells) in MSI CRCs were significantly negatively correlated with the SNV and indel AF variance (Spearman’s rank correlation test; Supplementary Fig. 9). There were fewer neutrophils in the less clonal MSI tumours (Fig. 4c; Spearman’s rank correlation test, *P* = 0.007 and 0.008 for indels and SNVs, respectively).

Somatic mutational signatures in cancer genomes reflect distinct mutational processes that have been operative throughout tumorigenesis, and several have been associated with defective DNA mismatch repair [56]. Correlation of a particular mutational signature in MSI CRCs with higher immune cell infiltration could imply a key role in the generation of neoantigens. We compared single base substitution (SBS), doublet base substitution (DBS) and indel (ID) signatures to T cell and myeloid cell infiltration across our MSI CRC sample set. None of the detected mutational signatures significantly correlated with T cell infiltration or the immune cell score, nor showed significant positive correlations to myeloid cell densities (Spearman’s rank correlation test; Supplementary Fig. 10).

The consensus molecular subtype (CMS) is a transcriptome-based robust classification system of CRCs with associations to tumour biology and prognosis [36]. Cytotoxic lymphocyte infiltration predicted from gene expression signatures was previously shown to be higher in CMS1 subtype MSI CRCs compared to CMS2-4 MSI CRCs [6]. The CMS was determined for the 43 MSI CRCs based on RNA sequencing data. Consistent with previous research [6, 36], the majority of the MSI tumours were classified into the MSI-associated CMS1 group (Fig. 4d). A substantial fraction of non-CMS1 tumours were also observed, yet these were not significantly overrepresented among the low immune cell score MSI tumours (all sporadic; Fisher’s exact test; Fig. 4d). Myeloid cell densities showed no significant correlations with CMS (Spearman’s rank correlation test; Supplementary Fig. 9).

### Expression of immune checkpoint molecules

**Fig. 5:**
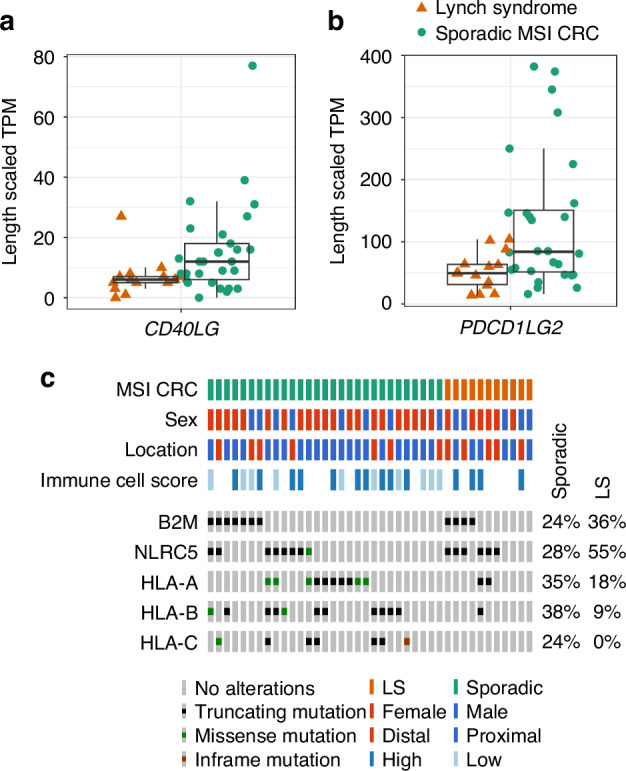
Immune evasion in LS and sporadic MSI CRCs. The expression of immune checkpoint molecules **a** CD40LG and **b** PDCD1LG2, in 29 sporadic and 14 LS tumours, represented as the length-scaled transcripts per million (TPM). **c** Non-synonymous mutations (SNVs and indels) in B2M, NLRC5 and HLA-A/B/C in 29 sporadic MSI and 11 LS tumours. Variant effect predictions required for the Oncoprint image were generated from BasePlayer somatic variant annotations [47]. The percentage of samples carrying a mutation is indicated on the right side.

The expression of multiple immune checkpoint molecules is upregulated in MSI CRCs as a mechanism of immune escape [11]. Variations in their expression between sporadic MSI and LS tumours could provide new insight into effective targeting of these tumours by immunotherapy. We compared the expression of 48 immune checkpoint molecules comprising both ligands and receptors [57–61] (Supplementary Table 3). Clustering of the tumours based on their expression did not lead to separation of sporadic and LS tumours, high immune cell score tumours, nor *BRAF-*mutant tumours (Supplementary Fig. 11). Three immune checkpoint ligands were significantly differentially expressed between the two tumour groups: *CD40LG, PDCD1LG2* and *CD70* (Deseq2, *P* = 2.1 × 10^−^^4^, 8.2 × 10^−^^4^ and 9.4 × 10^−^^4^, respectively; Fig. 5a, b, Supplementary Figs. 12 and 13). The differential expression of *CD70* was, however, driven by a single outlier. *CD40LG* expression positively correlated with infiltration of neutrophils and M2-like macrophages (Spearman’s rank correlation test with Bonferroni correction, adjusted *P* = 0.007 and 0.02, respectively), but not with T cell infiltration. *PDCD1LG2* expression did not show any correlations to immune cell infiltration.

Outlier samples in which we identified high expression of any of the 48 immune checkpoint proteins were typically only an outlier regarding the expression of a single gene, rather than tumours showing broad changes in immune checkpoint molecule expression.

### Somatic mutations in antigen presentation machinery (APM)

Antigen presentation via HLA complexes is essential for the recognition of tumour cells by T cells, and MSI CRCs display frequent mutations in genes related to HLA-mediated antigen presentation [23]. We analysed somatic non-synonymous mutations (SNVs and indels) in 30 genes involved in HLA class I antigen presentation (REACTOME pathway R-HSA-983170 and *NLRC5*, the key transcriptional activator of HLA class I genes) [62, 63]. Almost all (28/30) were mutated in at least one MSI CRC (Supplementary Fig. 14). *SEC31A*, *NLRC5*, *HLA-B*, *HLA-A*, *TAP2* and *B2M* were mutated most frequently when considering the sporadic MSI and LS tumours together. *B2M* and *HLA-A/B/C* encode the structural components of HLA class I molecules, while *NLRC5* induces their gene expression [64]. The vast majority, 86% of sporadic MSI and 64% of LS tumours, had a somatic mutation in at least one of these key genes with no obvious correlation to immune cell score (Fig. 5c). *HLA-A/B/C* mutations were less common in LS tumours (18% vs 66% of sporadic MSI tumours).

Considering all somatic non-synonymous variants (SNVs and indels), only the COP II complex gene *SEC31A* was significantly more often mutated in sporadic MSI compared to LS tumours (Fisher’s exact test, *P* = 0.027); a similar trend was seen for other COP II complex members (Supplementary Fig. 15) [65]. *SEC31A* also had significantly more indels, and *HLA-A* had more SNVs in sporadic MSI tumours (Fisher’s exact test, *P* = 0.03 and 0.043), while *NLRC5* had more indels in LS tumours (*P* = 0.015). None of the 30 HLA class I genes were differentially expressed at the mRNA level between sporadic MSI and LS tumours, or between their normal tissues.

CRC cells have also been found to express HLA class II complexes on their cell surface [66]. Among 130 genes related to HLA class II antigen presentation (REACTOME pathway R-HSA-2132295 and *CIITA*, the key transcriptional activator of HLA class II genes) [67], only one was significantly differentially mutated between LS and sporadic MSI tumours: *SEC31A* (also involved in the HLA I pathway; Supplementary Fig. 16).

### Neoantigen burden

**Fig. 6:**
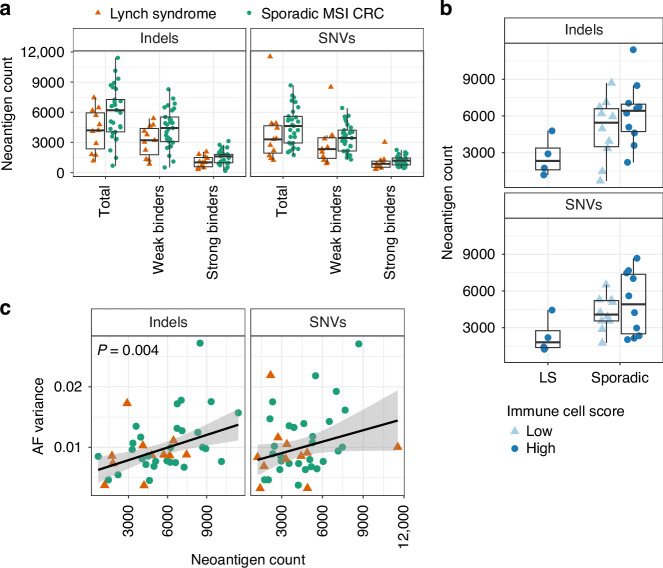
The predicted neoantigens in 11 LS and 29 sporadic MSI CRCs. **a** The raw counts of the total, weak-binding and strong-binding neoantigens. **b** The total neoantigen count in high and low immune cell score tumours. **c** The allelic fraction (AF) variance of indels and SNVs compared to the total neoantigen count. Statistically significant correlations are indicated by the *P*-value. Other differences are not statistically significant.

The high mutation burden of MSI CRCs translates to a large number of neoantigens being presented by the HLA complexes. Using somatic variant calls from WGS data, we compared the number of neoantigens predicted to bind HLA class I molecules in LS and sporadic MSI CRCs using the NeoPredPipe tool [37]. There were no statistically significant differences in the raw counts of total neoantigens, or those predicted to bind weakly or strongly to HLA molecules (Fig. 6a). The trend towards higher neoantigen burden observed in sporadic MSI tumours was partially explained by the trend towards higher total variant counts (Supplementary Fig. 17, normalised counts), as these showed a positive correlation (Linear regression, *P* = 2 × 10^−^^9^ and *P* = 5.6 × 10^−^^19^ for the total neoantigen counts vs total indel and SNV counts, respectively). Interestingly, there was no significant correlation between the number of predicted neoantigens and the immune cell score (Fig. 6b) or raw T cell densities (Supplementary Fig. 18). Similarly, the presence of a *BRAF* V600E mutation in the tumour did not predict the neoantigen count (Supplementary Fig. 19). The neoantigen burden showed, however, a significant positive correlation with AF variance (Linear regression, *P* = 0.004 for the total indel neoantigen count vs indel AF variance; Fig. 6c selected panels, Supplementary Fig. 20). This suggests a higher neoantigen burden in tumours with low clonality; in our cohort, these are almost exclusively sporadic MSI tumours [35]. Neutrophils tend to be excluded from these tumours with low clonality, as AF variance correlated negatively with neutrophil infiltration (Fig. 4c).

## Discussion

The highly immunogenic environment of MSI CRC makes it particularly responsive to immunotherapy, yet at least half of tumours still show a lack of response [20]. In addition to immune checkpoint inhibitors already in clinical use, there are many other potential drug targets for immunotherapy under consideration [68]. A detailed understanding of differences in the immune contexture of tumours can lead to insight as to how immunotherapy can be used most effectively in patients. The purpose of this study was to compare the innate and adaptive immune microenvironment and immune evasion mechanisms of sporadic MSI and LS CRCs using a multi-layered dataset comprising immunohistochemical detection of both T cells and myeloid immune cells, WGS and RNA-seq from the same set of tumours to enable cross-comparisons.

We found higher immune cell scores, depicting higher T cell infiltration, in MSI CRCs compared to MSS CRCs, as expected [5, 32]. Not all MSI CRCs had a high immune cell score of 3–4, as also reported in other cohorts [14]. All our LS tumours had high immune cell scores, compared to approximately half of the sporadic MSI tumours. A previous comparison of the immune cell scores in a larger cohort identified no significant difference between sporadic MSI and LS CRCs [32]. Nevertheless, when analysing raw T cell densities, several previous studies have identified significantly higher T cell infiltration in LS tumours compared to sporadic MSI CRCs [29, 31, 69], compatible with our results; while some studies found no significant difference, no studies reported higher T cell density in sporadic MSI CRCs [69]. The variability we observed in the immune cell scores of sporadic MSI tumours could be associated with further underlying heterogeneity in the immune response, such as the contribution of natural killer cells [70], or myeloid immune cell subsets as revealed by our data.

We showed here that T cell densities significantly correlated with the densities of several monocytic, but not with any of the granulocytic cell subsets, across our MSI CRC cohort. This suggests a stronger interdependence between the T cell-mediated adaptive antitumour response and the innate response mediated by cells of the monocyte-macrophage lineage. We found significantly higher overall density of M2-like macrophages in sporadic MSI CRCs compared to LS tumours, as well as lower M1/M2 macrophage ratio in the invasive margin stroma. M2-like macrophages have known immunosuppressive and protumorigenic functions that may hamper the antitumour T cell response in sporadic MSI tumours [24, 26, 71]. Importantly, M2-like macrophages augment drug resistance and facilitate tumour metastasis [71]. Both high M2-like macrophage cell counts and a low M1/M2 ratio in the invasive margin have been correlated with lymph node metastasis in CRC [72]. The differing macrophage landscapes in sporadic MSI and LS tumours may thus have implications in metastasis, and are compatible with the poorer overall survival of sporadic MSI CRC patients [73, 74]. Numerous therapeutic approaches to target tumour-associated M2-like macrophages are currently under development, and these are increasingly entering clinical trials [24, 71].

We found heterogeneity in tumour-associated macrophages also within the sporadic MSI tumour group. Tumours with a high immune cell score (high T cell infiltration) showed significantly increased infiltration of M1-like macrophages compared to those with a low immune cell score, while M2-like macrophage densities were similar. This is in line with the proinflammatory functions of M1-like macrophages that reinforce the antitumour immune response [26] and implies that differences in macrophage polarisation states may contribute to the observed heterogeneity in T cell infiltration among sporadic MSI CRCs. This heterogeneity in the immune landscape could have an effect on immunotherapy response. A recent report found that among MSI CRC patients, *BRAF* mutations in sporadic tumours were associated with poorer overall survival after immunotherapy, while the survival outcomes were similar in sporadic MSI and LS tumours [75]. An earlier study focusing on metastatic MSI CRC showed opposite results, finding no association between *BRAF* V600E mutations and survival, while showing improved survival in immune checkpoint inhibitor-treated patients with LS-associated compared to sporadic MSI tumours [76]. In our sporadic MSI tumours, non-metastatic with the exception of one tumour [35], *BRAF* mutation status was not associated with the differences in T cell infiltration.

The stimulatory *CD40LG* and inhibitory *PDCD1LG2* immune checkpoint molecules were found to be more highly expressed in sporadic compared to LS MSI CRCs. High *CD40LG* expression is present in activated CD4+ T cells, and it acts via the CD40 receptor to activate B cells and myeloid cells [77]. Several agonistic anti-CD40 monoclonal antibodies are undergoing clinical testing in patients with solid tumours with the rationale of activating antigen presentation by dendritic cells and tumoricidal activity associated with M1-like macrophages [24, 78, 79]. However, we found a positive correlation between *CD40LG* expression and M2-like macrophage densities, which warrants further study. *PDCD1LG2* (PD-L2) is a ligand to PD-1 alongside the better-known PD-L1, and shares a closely related T cell inhibitory function with these key immunotherapy targets [80]. Both *PD-L1* and *PD-L2* are upregulated in MSI vs MSS CRCs [81], and *PD-L2* expression independently associates with poor survival in CRC [82]. Interestingly, immunohistochemical stainings across multiple cancer types suggested high *PD-L2* expression as a potential biomarker for cancer types with low response rates to immune checkpoint blockade [83]. Thus, elevated *PD-L2* expression could modulate immunotherapy response in sporadic MSI CRCs and provide an additional target for therapy [81, 83].

In previous studies, HLA class I loss at the protein level was observed more frequently in sporadic MSI tumours and driven by a broader spectrum of mutations, while *B2M* mutations are more common in LS MSI CRC [69, 84]. These trends were also visible in our cohort. We found a significantly higher proportion of indels in *NLRC5* in LS CRC. *NLRC5* is frequently mutated in MSI CRC, and is the most commonly altered target among HLA I pathway genes across cancer types [85, 86]. *NLRC5* transcriptionally activates HLA class I genes along with other key genes in the pathway [64], thus having a broad impact on HLA I pathway function similar to *B2M*. Sporadic MSI CRCs carried more SNVs and indels in *HLA-A* and *SEC31A*, respectively. Overall, we found only subtle differences between sporadic MSI and LS tumours in immune evasion via mutations in HLA class I and class II pathway genes.

An earlier study found increased neoantigen burden in LS compared to sporadic MSI CRCs [29], while we found no significant difference. The study used exomes compared to our whole genomes and a different pipeline for neoantigen prediction, which may have contributed to the differing results. Heterogeneity among the sporadic MSI tumours may also have played a role. We previously showed in our cohort that sporadic MSI CRCs are, on average, less clonal compared to LS tumours, and display higher variation in clonality, indicated by the AF variance proxy [35]. Here, we found a significant correlation between neoantigen burden and tumour clonality in the same cohort of MSI CRCs; less clonal tumours had a higher neoantigen burden. Thus, the subset of sporadic MSI tumours showing the highest clonal heterogeneity is associated with elevated numbers of subclonal neoantigens. Surprisingly, we found no correlation between neoantigen burden and T cell infiltration in MSI CRCs, despite the key role of frameshift neoantigens in triggering antitumour T cell responses [2–5, 34]. Consistent with our results, analyses of 333 MSI CRCs across multiple cohorts revealed that total mutation load correlated with the number of predicted neoantigens, but neither correlated with cytotoxic lymphocyte infiltration estimated from transcriptomic data [6]. A recent landmark study in a mouse model of sporadic MSI CRC offers an intriguing explanation, demonstrating that T cell responses against neoantigens were attenuated with decreasing neoantigen clonality [87]. The study further showed that clonal, but not subclonal, neoantigen burden predicted response to immune checkpoint blockade in clinical trials of metastatic MSI colorectal and gastric cancer [87]. Our findings suggest that this phenomenon could contribute to the observed differences in tumour immune contexture between sporadic and LS-associated MSI CRCs, as well as to heterogeneity among the sporadic MSI tumours.

This study was limited by the small tumour sample size, particularly of LS samples, given their low incidence at a population level, and an inability to perform all analyses for all tumours. Almost all patients in the MSI CRC cohort analysed here were non-metastatic [35], and thus, implications for metastatic tumours may be less direct. The immune milieu in non-metastatic and metastatic MSI CRC may be very different, as suggested by differences in clinical responses to immunotherapy [15–17, 20]. All LS tumours included in this study were *MLH1*-defective, while LS tumours defective in *MSH2, MSH6* or *PMS2* were not represented. While this selection allowed a more straightforward comparison with sporadic MSI, our findings may not generalise to all LS patients. It would be beneficial to expand future studies to LS patients with defects in other MMR genes. Additionally, we only sampled a single section of the tumour at a single point in time. As a consequence, we have only a limited snapshot of the highly dynamic tumour immune microenvironment. The complexity of the immune system means that some assumptions are required in selecting cell markers. In future studies, it would be interesting to consider additional cell types such as NK cells, dendritic cells, B cells or myeloid-derived suppressor cells, along with more specific subtypes of cell lineages we did include.

Taken together, our findings reveal differences in both the T cell and myeloid immune cell landscapes between sporadic MSI and LS tumours. All LS tumours showed a high immune cell score, depicting high T cell infiltration, compared to only half of sporadic MSI CRCs. M2-like macrophages, associated with immunosuppressive and protumorigenic functions [24, 26], were more prevalent in sporadic MSI tumours. Sporadic MSI tumours also expressed more PD-L2, an immunosuppressive PD-1 ligand involved in the PD-1/PD-L1 signalling axis targeted by current CRC immunotherapy regimens. We also identified heterogeneity within the sporadic MSI tumours. Only half of them presented with a highly T cell-infiltrated microenvironment associated with increased M1-like macrophage densities. We found an inverse correlation between tumour clonality and neoantigen burden in MSI CRCs. MSI tumours with low clonality are associated with a high burden of subclonal neoantigens, which could blunt the immune response in a subset of sporadic MSI CRCs [87]. Collectively, these factors may all contribute to the variable responses observed among MSI CRC patients to current immune checkpoint therapies [15–17, 20].

There is still much to be learned about the role of the immune system in CRC, and, in particular, the innate immune branch, which is often overlooked in favour of the T cell infiltration more extensively studied in cancers. A more complete understanding of the differences in immune landscape and immune evasion tactics between sporadic MSI and LS CRCs could allow recognition of specific targets for the design of more effective immunotherapy regimens for each tumour subgroup. More broadly, insights gained from MSI CRCs could also provide clues for identifying subgroups of MSS CRCs that could benefit from immunotherapy.

### Reporting summary

Further information on research design is available in the Nature Research Reporting Summary linked to this article.

## Supplementary information

Supplementary information

Reporting Summary

## Data Availability

Somatic calls from MSS CRCs are available on the EGA database (Accession code EGAS00001004710). Additional somatic data is available on request from the corresponding author.
