# Supplementary material for: Tumour immune contexture and immune evasion in sporadic and Lynch syndrome-associated microsatellite unstable colorectal cancers
Source: Br J Cancer. 2026 Jan 14;134(7):1019–30. doi: 10.1038/s41416-025-03302-z (PMC12996609; doi:10.1038/s41416-025-03302-z)
Supplement: Supplementary file 1 — Supplementary information [file 41416_2025_3302_MOESM1_ESM.docx]

## Supplementary Figures

*
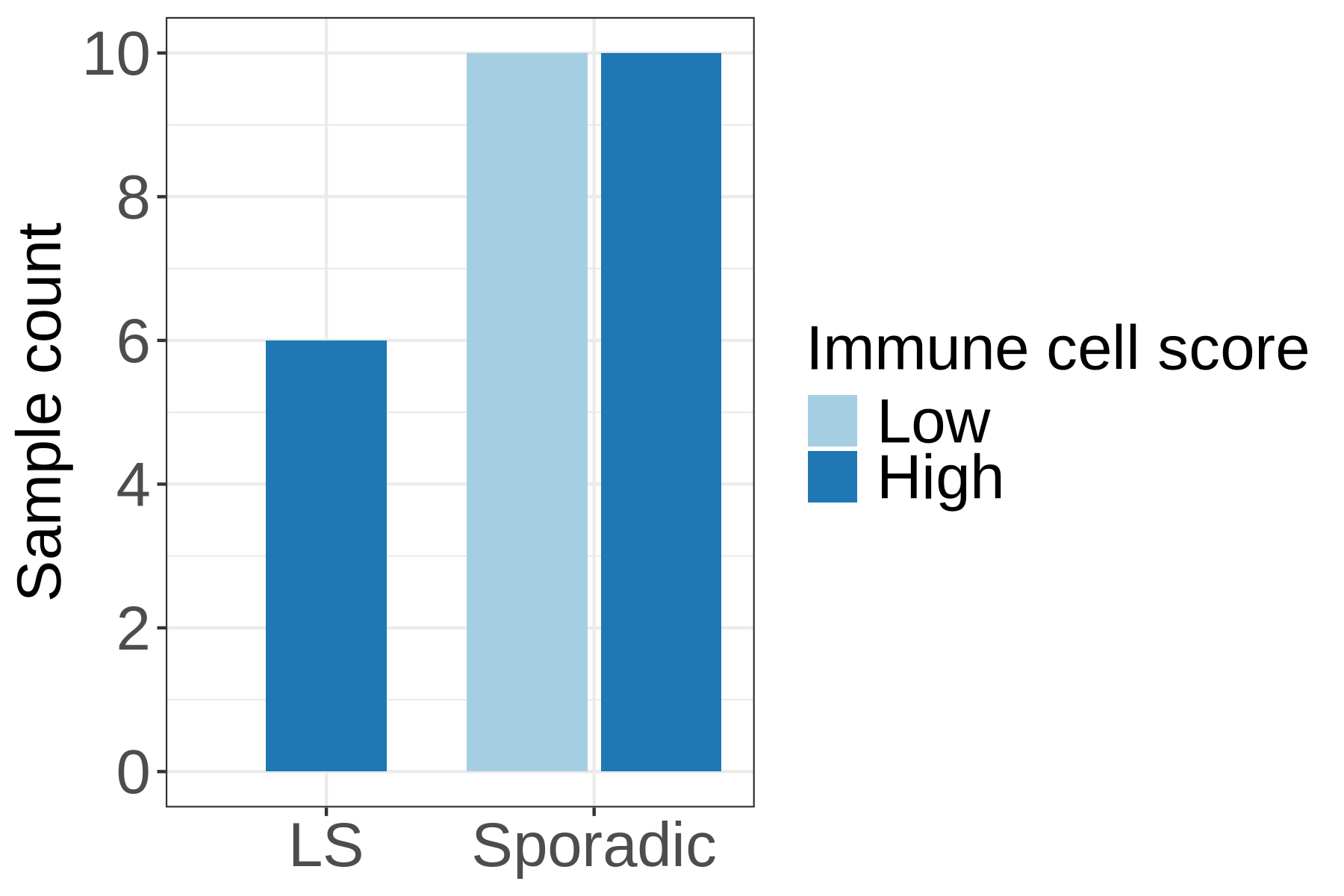
*

*Supplementary figure 1: Low (0-2) vs high (3-4) immune cell scores in 6 LS and 20 sporadic MSI CRCs.*

*
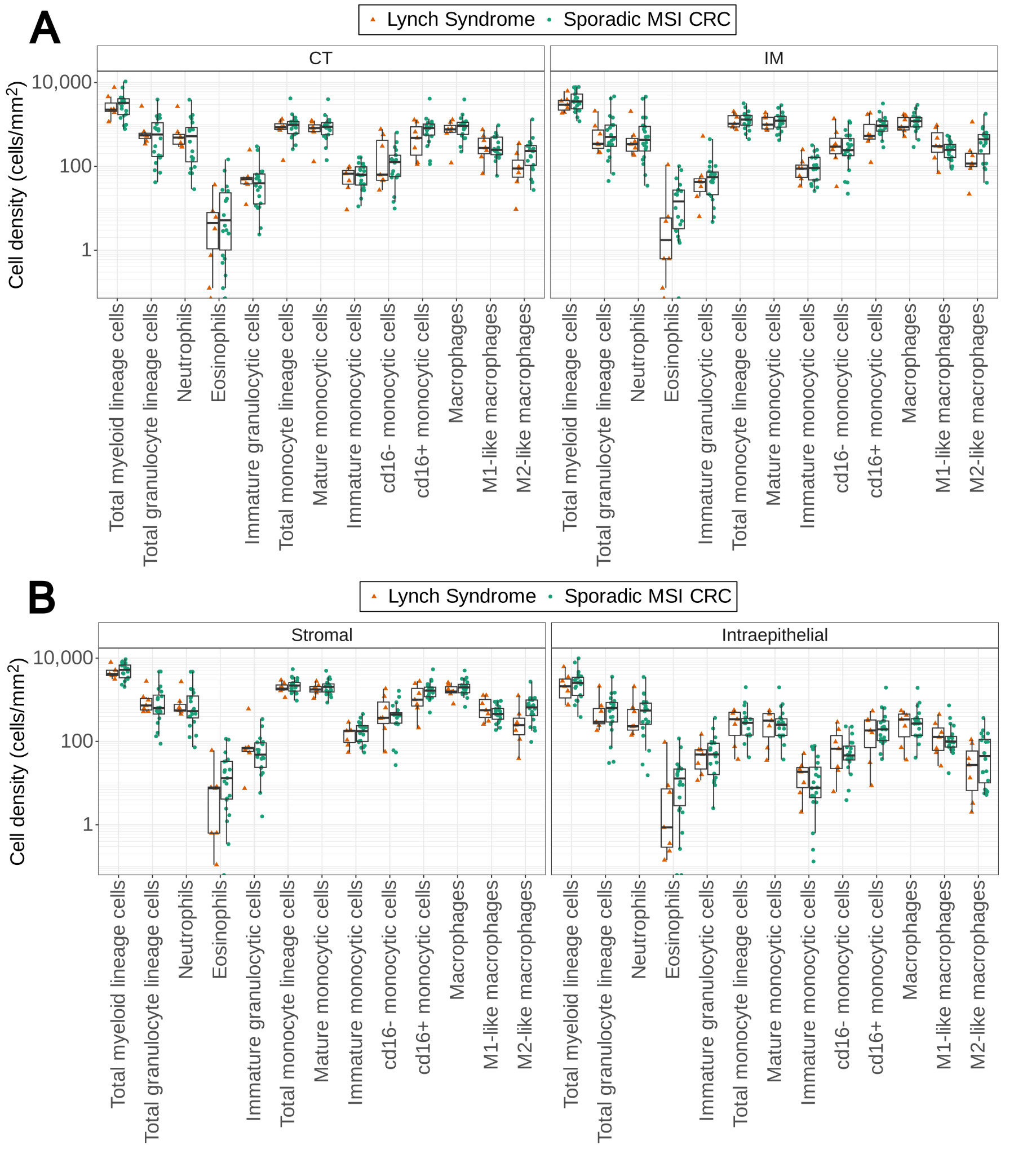
*

*Supplementary figure 2: Myeloid immune cell densities in 7 LS and 20 sporadic MSI tumours. Immune cell densities in (A) the tumour centre (CT) and invasive margin (IM), and (B) stromal and intraepithelial locations in the tumour. The y-axis is on a log scale*.


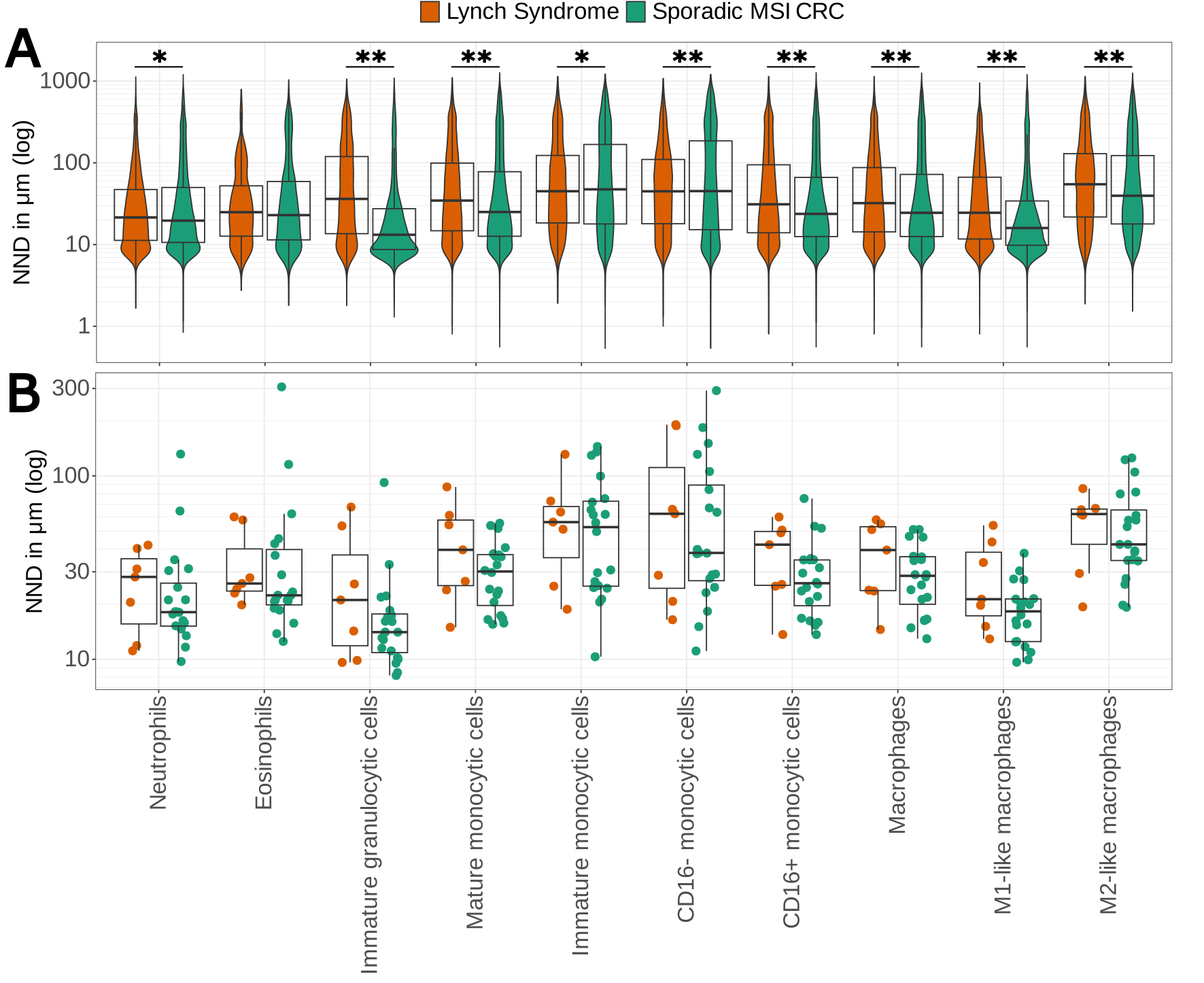


*Supplementary figure 3: The nearest neighbour distance of myeloid immune cell subsets to the nearest tumour cell in LS and sporadic MSI tumours. (A) Each individual cell across all tumour samples. (B) The median NND in each tumour sample. Statistically significant differences between sporadic MSI and LS are indicated by an asterisk (* P<5x10^-5^, ** P<5x10^-25^).*


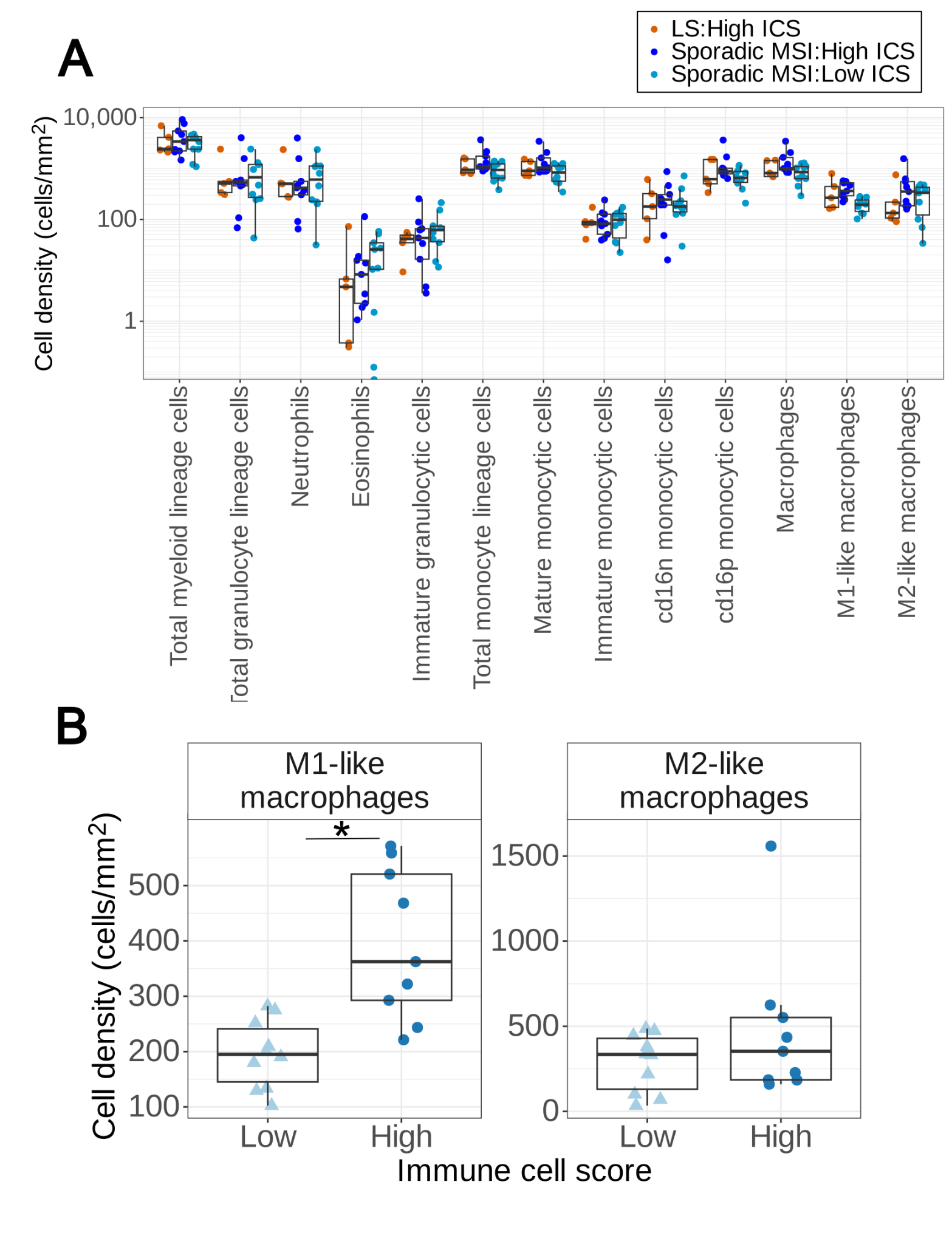


*Supplementary figure 4: Myeloid immune cell densities vs T cell immune cell score (ICS). (A) The densities of myeloid immune cells in high and low immune cell score tumours separated by sporadic and LS tumour status. (B) The density of M1-like and M2-like macrophages in high and low immune cell score sporadic MSI tumours. Statistical significance is indicated by the asterisk (*; Mann Whitney U test, P=6x10^-4^).*

*
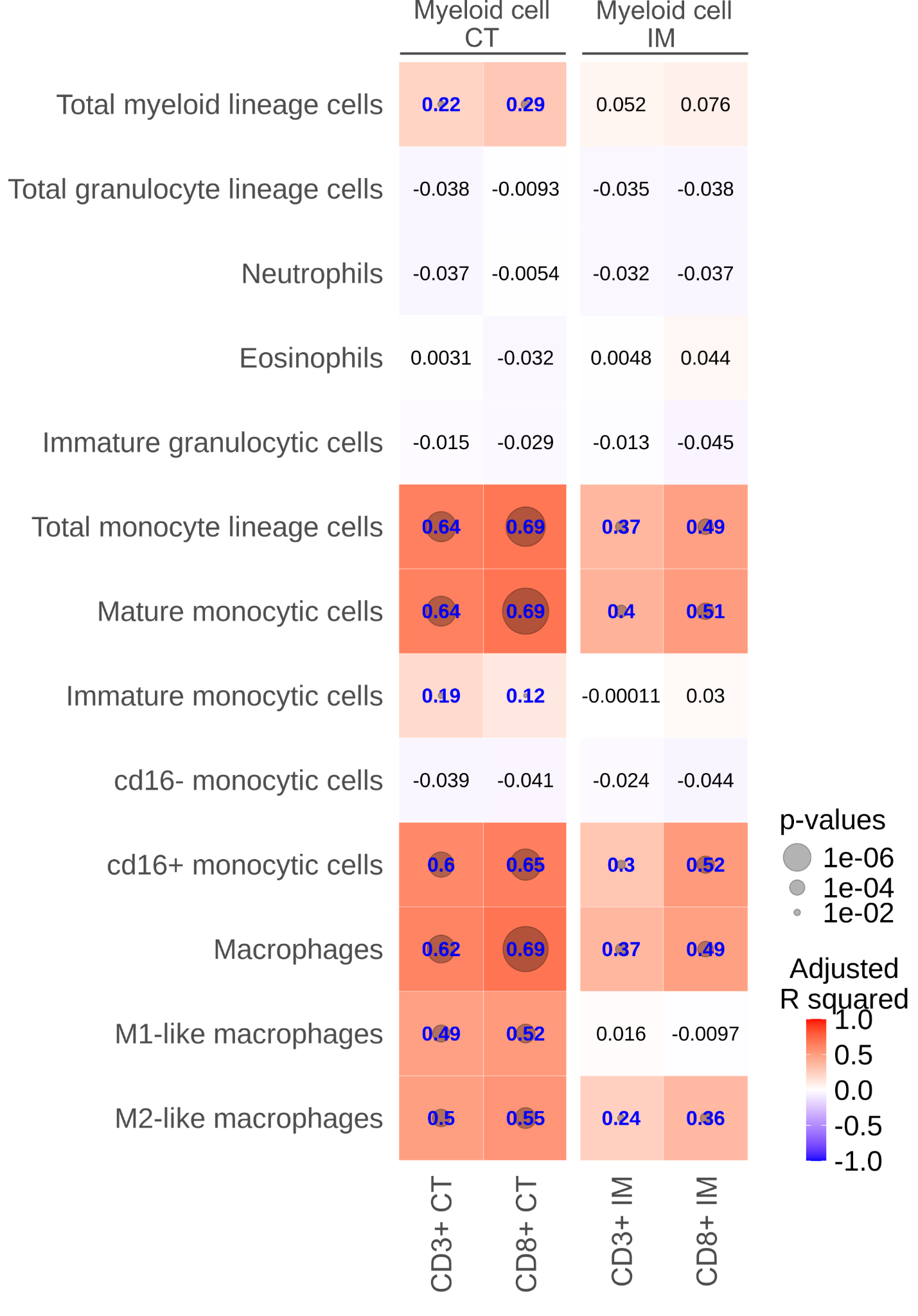
*

*Supplementary figure 5: Linear regression between infiltrating myeloid immune cell and T cell counts in (A) invasive margin (IM) and (B) tumour centre (TC) regions. Adjusted R squared values in bold blue text were significant (P<0.05).*

*
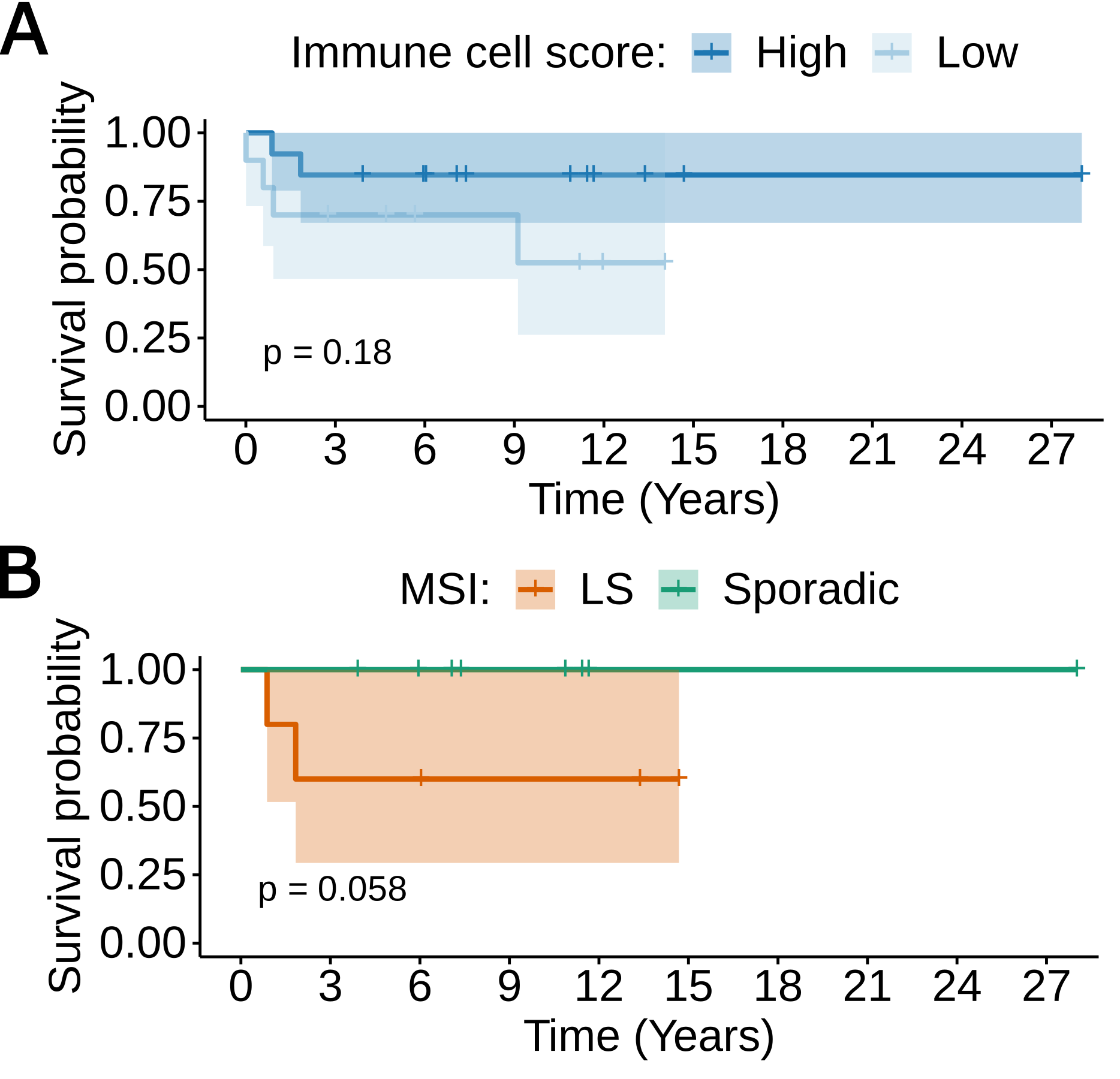
*

*Supplementary figure 6: CRC-specific survival of (A) 13 high vs 10 low immune cell score tumours including 5 LS and 18 sporadic MSI CRCs. (B) 5 LS vs 8 sporadic MSI CRCs with high immune cell scores. Tumours with missing cause of death information were excluded.*


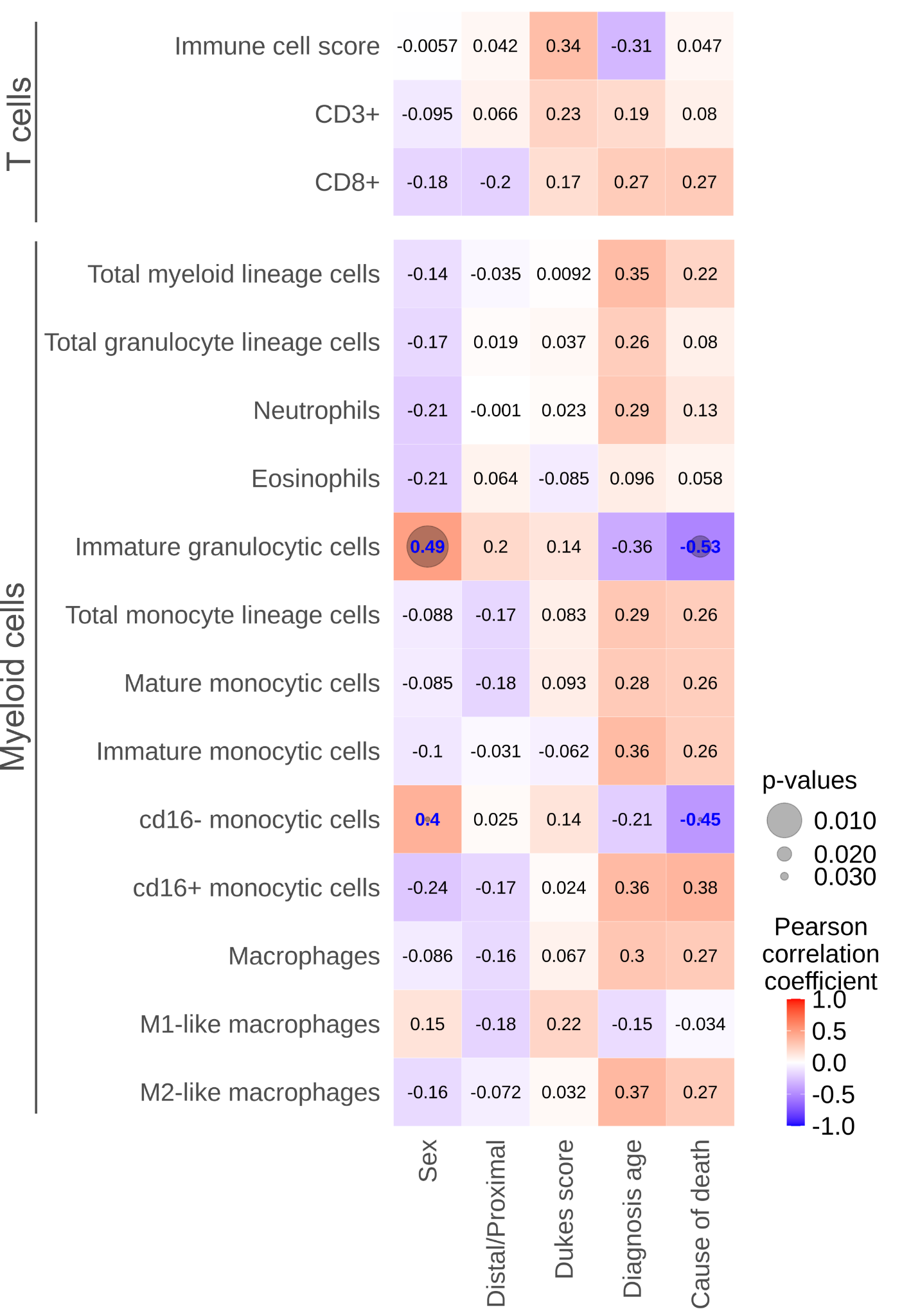


*Supplementary figure 7: Pearson's correlation estimates between T cell and myeloid immune cell densities and clinical factors Estimates in bold blue text were significant (P<0.05).*

*
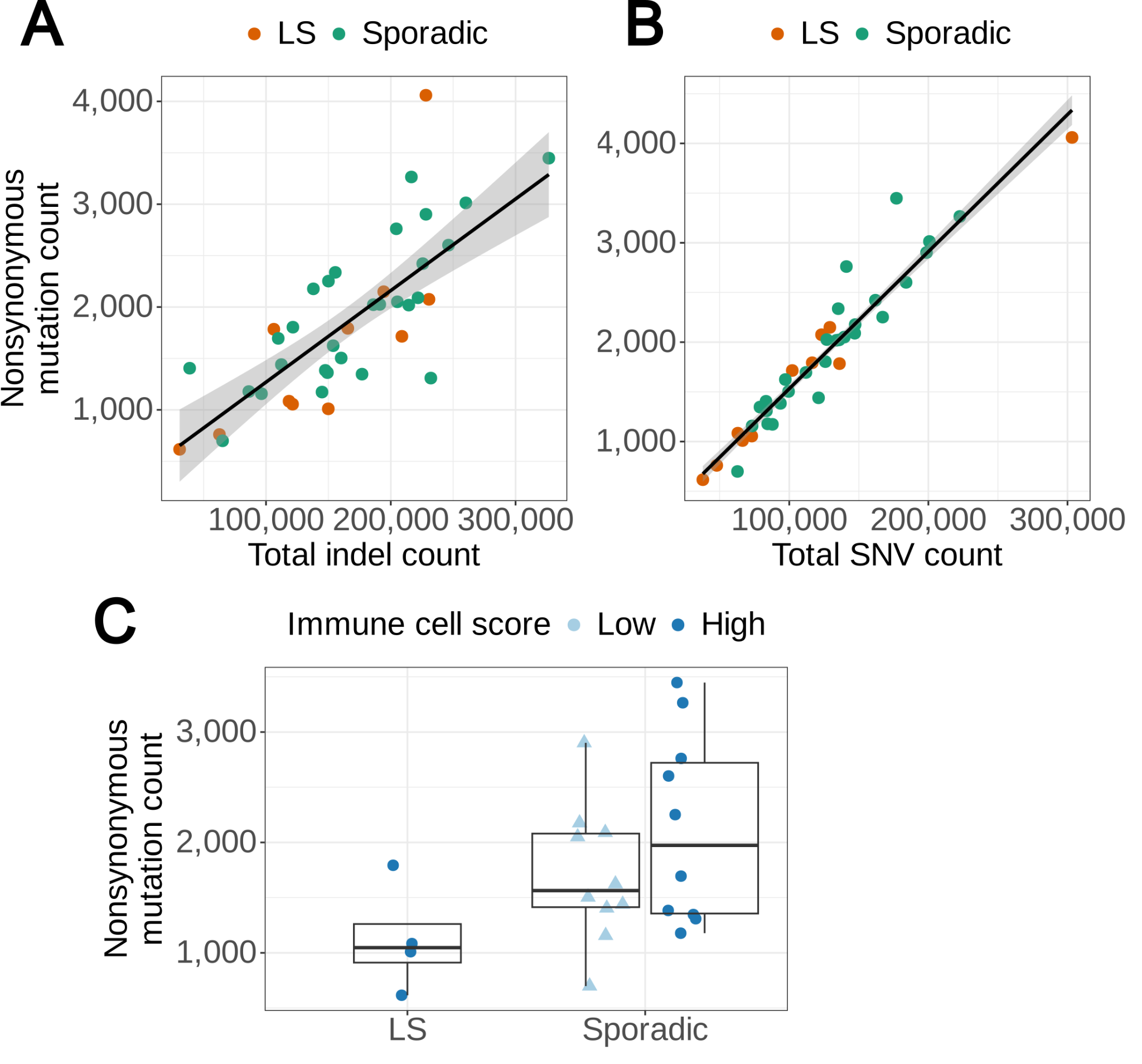
*

*Supplementary Figure 8: The number of non-synonymous mutations compared to the genome-wide counts of (A) indels and (B) SNVs. (C) The number of non-synonymous mutations in high and low immune cell score tumours in 4 LS and 20 sporadic MSI CRCs.*

*
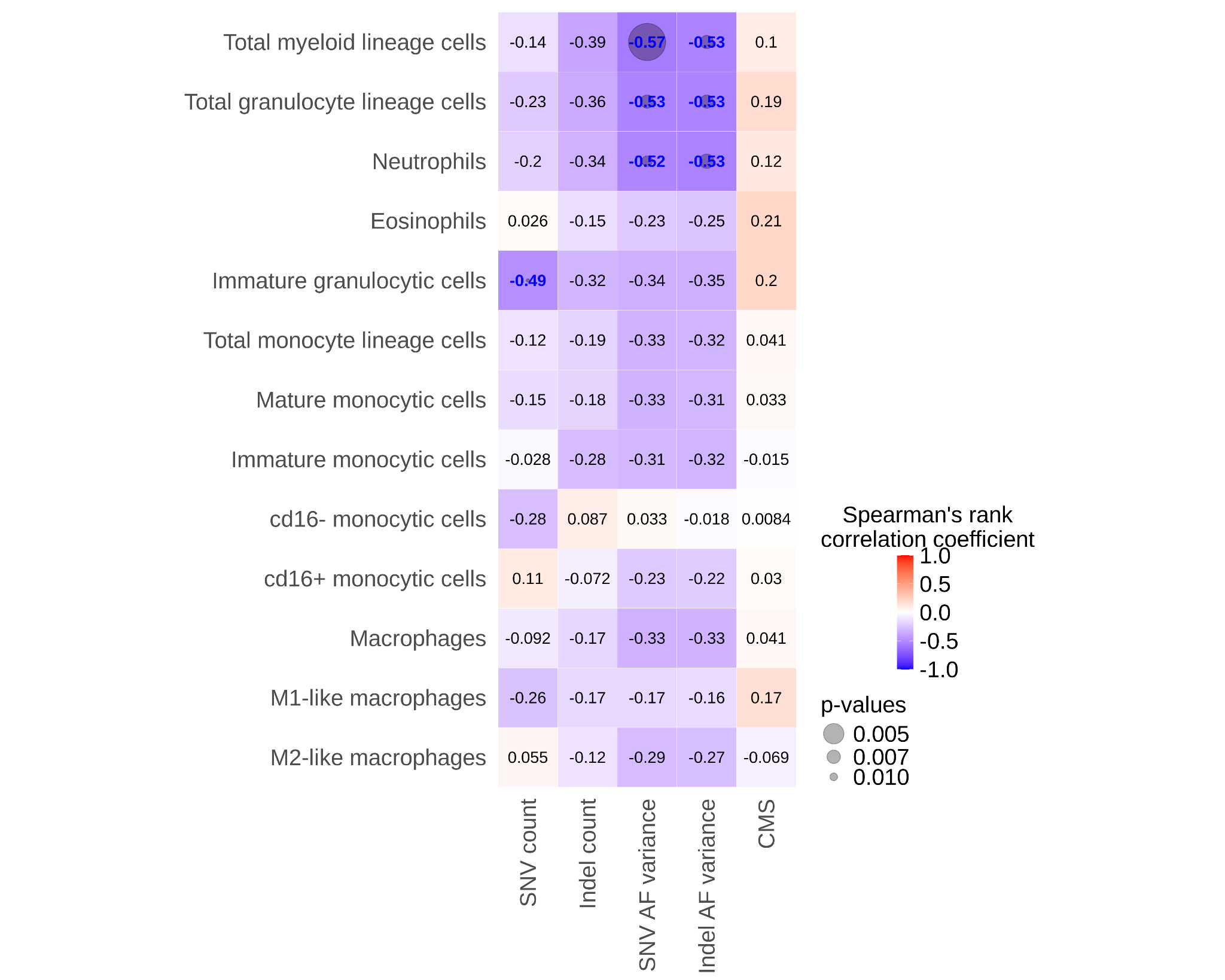
*

*Supplementary figure 9: Spearman's rank correlation estimates between myeloid immune cell infiltration, and somatic mutation counts, allelic fraction (AF) variances and the consensus molecular subtypes (CMS). Estimates in bold blue text were significant (P<0.05).*


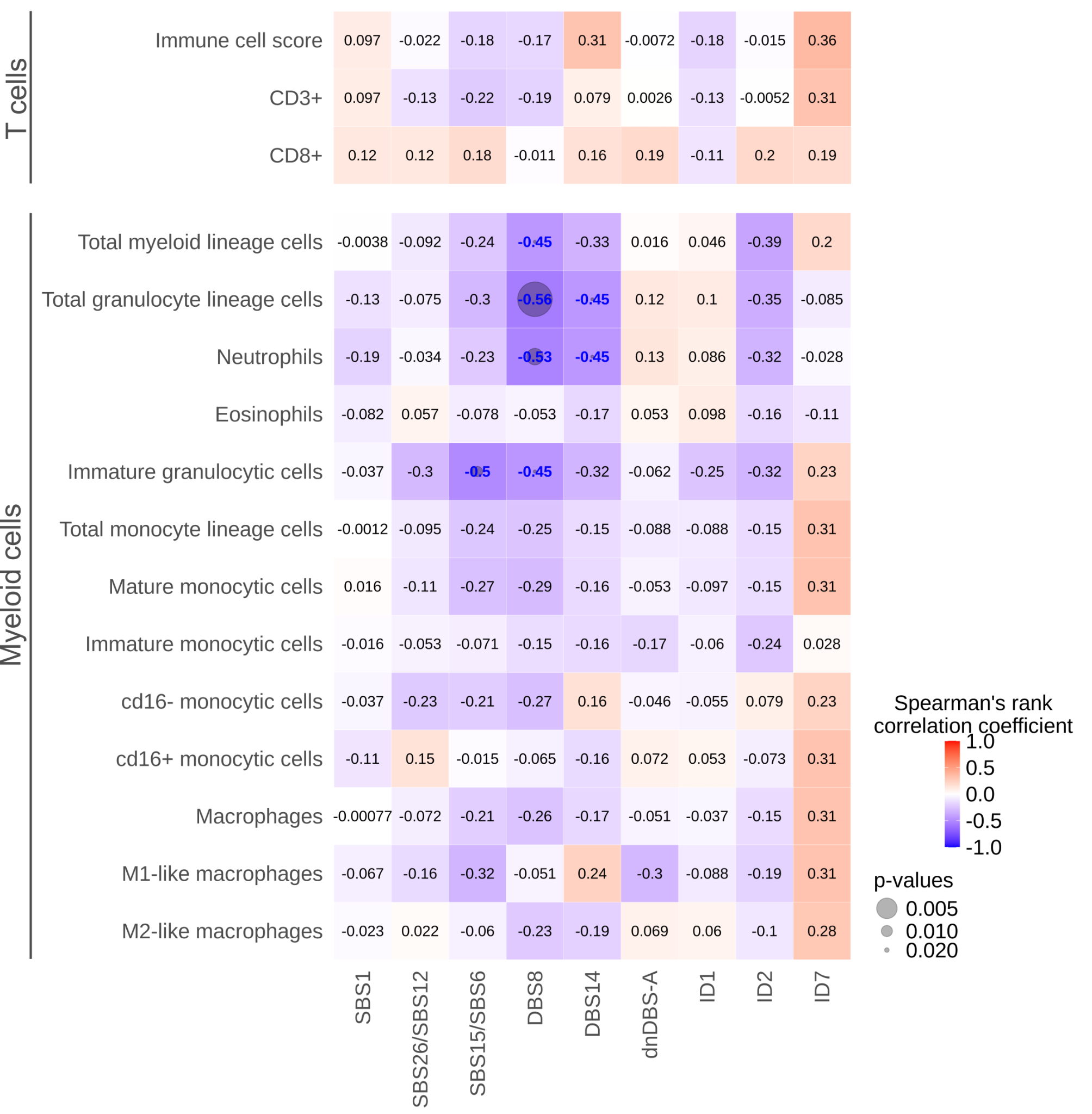


*Supplementary figure 10: Spearman's rank correlation estimates between T cell and myeloid immune cell infiltration, and single base substitution (SBS), doublet base substitution (DBS) and indel (ID) signatures. Estimates in bold blue text were significant (P<0.05).*

*
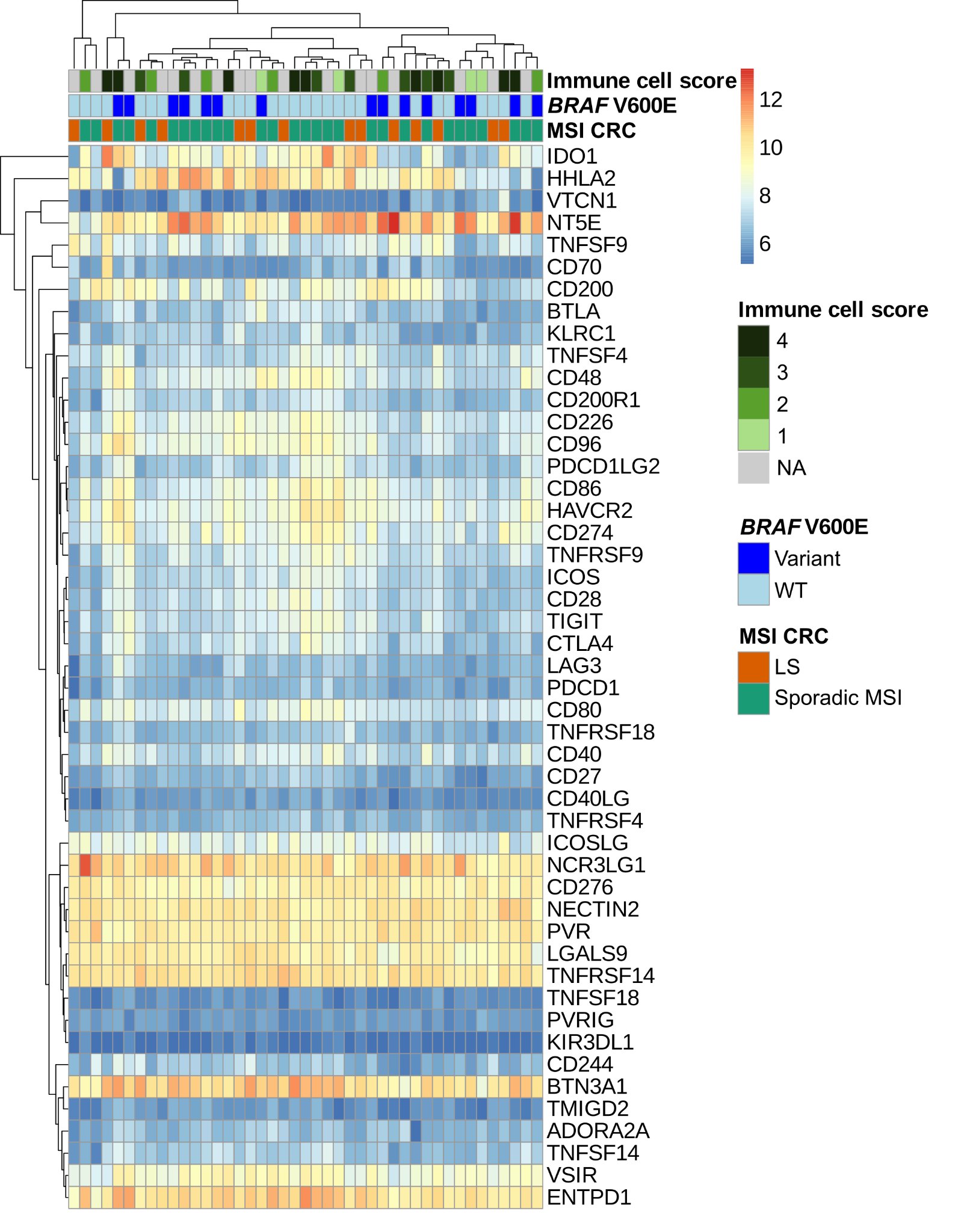
*

*Supplementary figure 11: Clustering of 48 immune checkpoint molecules in 29 sporadic and 14 LS tumours.*

*
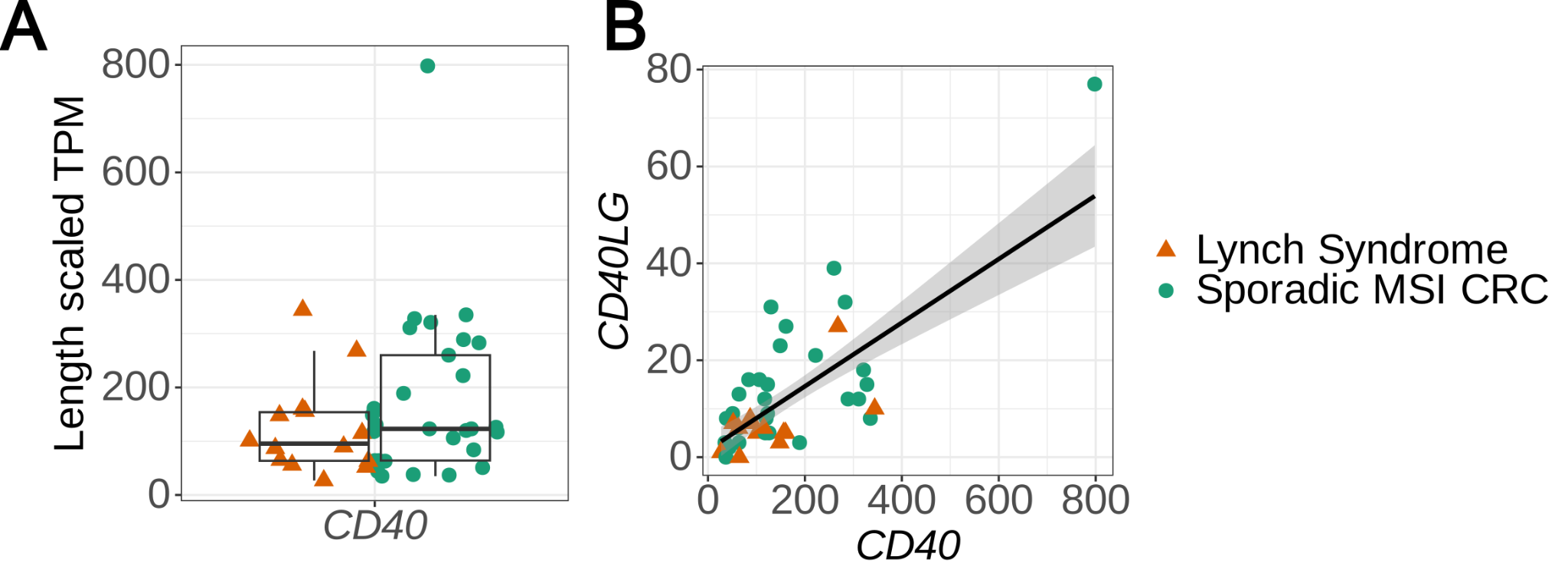
*

*Supplementary figure 12: The gene expression of CD40, the immune checkpoint receptor of ligand CD40LG in (A) LS vs sporadic MSI CRC and (B) compared to CD40 expression with a robust linear regression model fitted.*

*
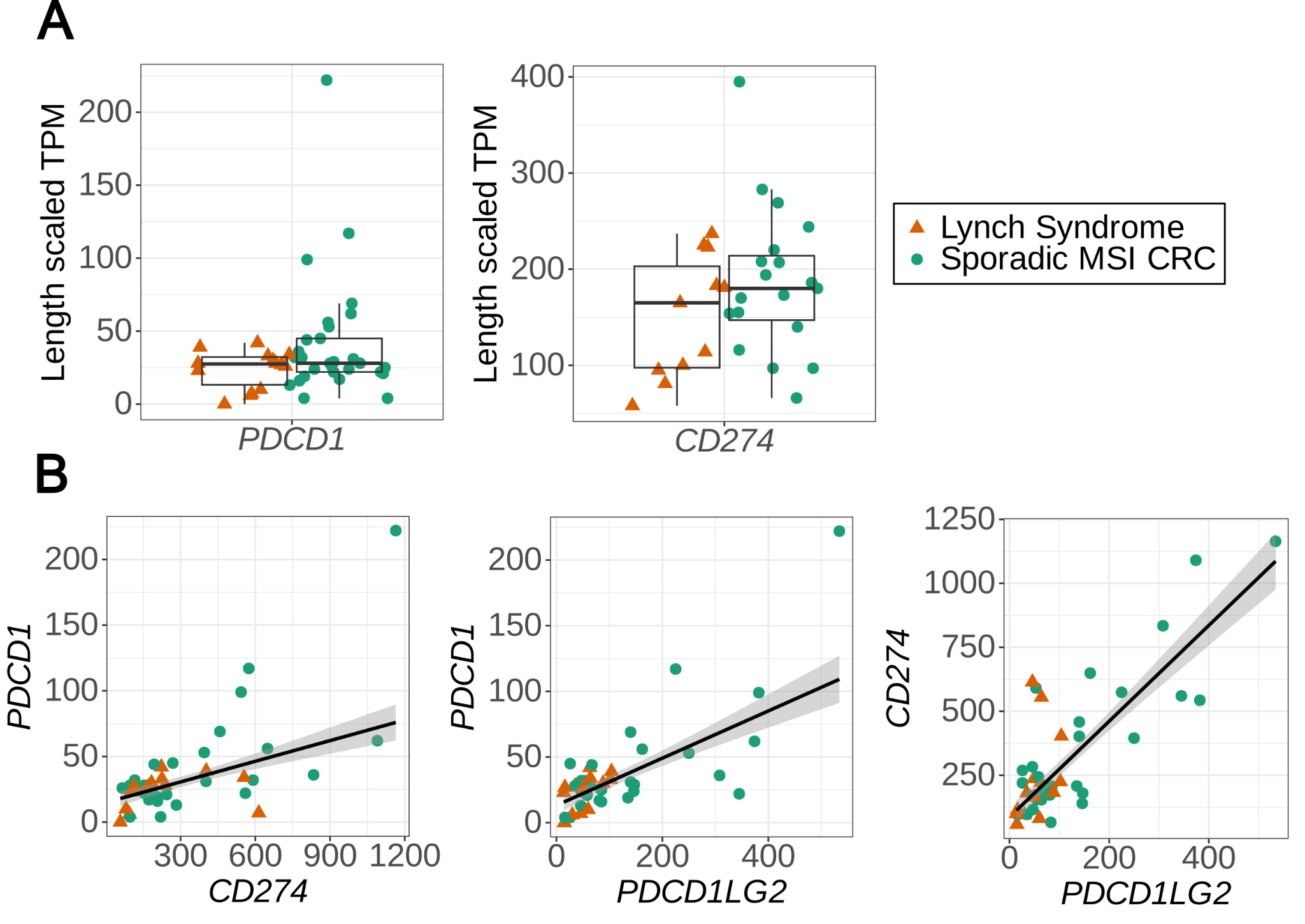
*

*Supplementary figure 13: Gene expression of PDCD1, the immune checkpoint receptor of ligand PDCD1LG2, and CD274, an additional ligand of PDCD1. In 29 sporadic MSI and 11 LS tumours. (A) Comparison of expression in sporadic MSI and LS tumours (B) Comparison of the gene expression of PDCD1 and its ligands PDCD1LG2 and CD274 in each MSI CRC with a robust linear regression model fitted.*

*
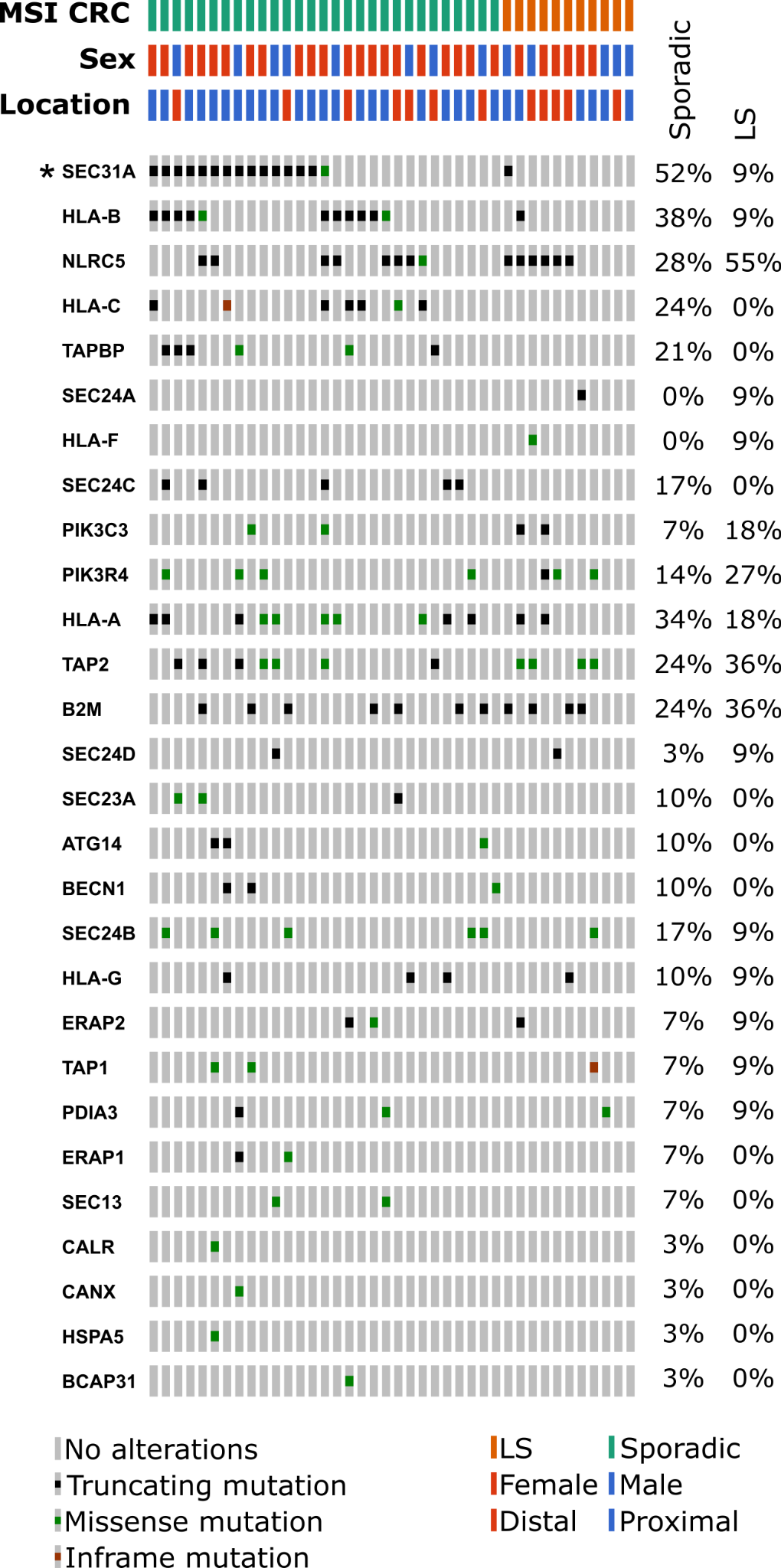
*

*Supplementary figure 14: Somatic non-synonymous mutations (SNVs and indels) in HLA class I antigen presentation-related genes in 29 sporadic MSI and 11 LS tumours. Genes are ranked by the differential variant frequency in sporadic MSI and LS indicated by Fisher's test P-values (* indicates the significant P-value, P=0.027). Variant effect predictions required for the Oncoprint image were generated from BasePlayer somatic variant annotations* [*(Katainen et al. 2018)*](https://paperpile.com/c/2Mn114/W0UO)*. The percentage of samples carrying a mutation is indicated on the right-hand side.*


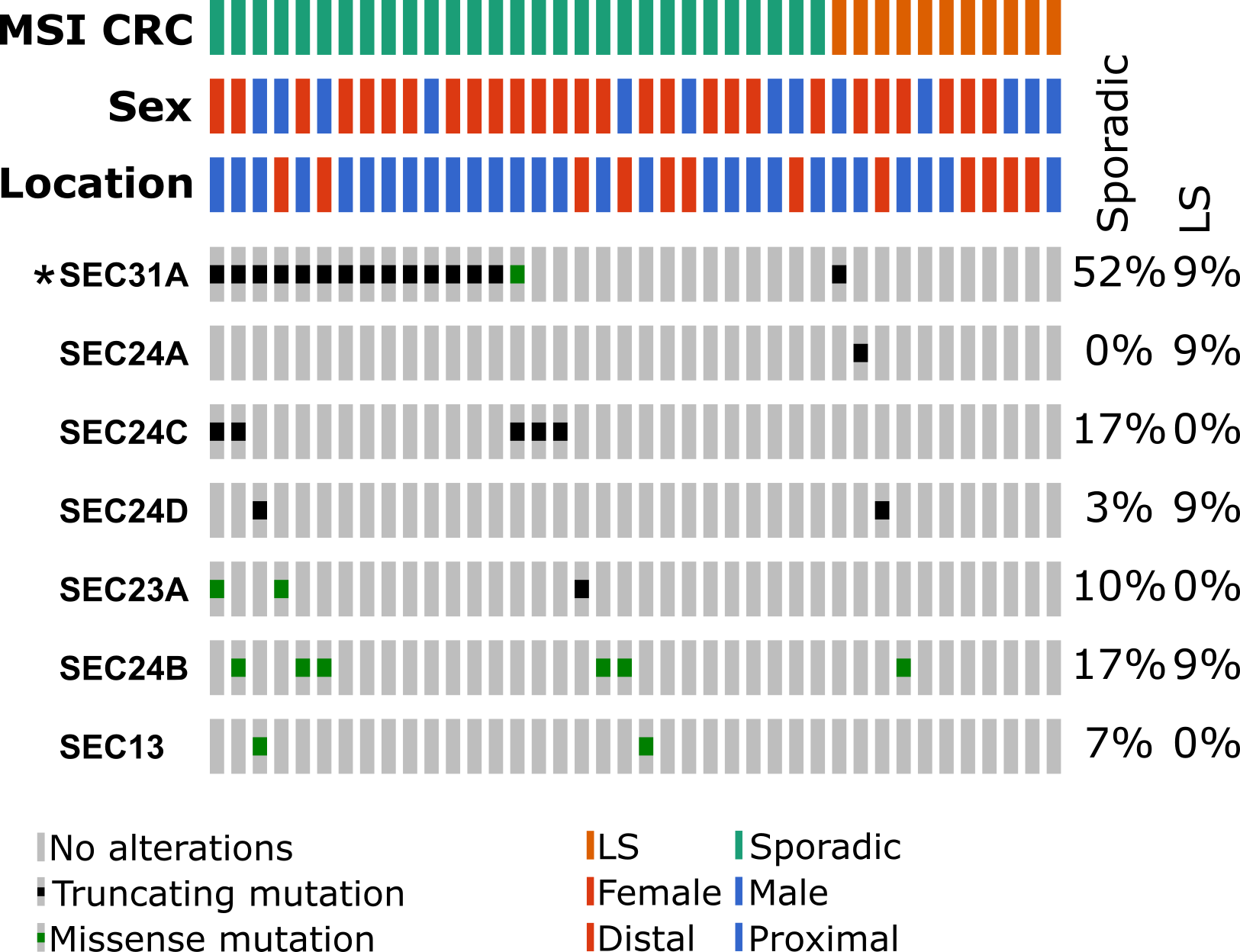


*Supplementary figure 15: Somatic non-synonymous mutations (SNVs and indels) in the COPII complex-related proteins among the 30 HLA class I proteins, in 29 sporadic MSI and 11 LS tumours. Genes are ranked by the differential variant frequency in sporadic MSI and LS indicated by Fisher's test P-values (* indicates the significant P-value, P=0.027). Variant effect predictions required for the Oncoprint image were generated from BasePlayer somatic variant annotations* [*(Katainen et al. 2018)*](https://paperpile.com/c/2Mn114/W0UO)*. The percentage of samples carrying a mutation is indicated on the right-hand side.*


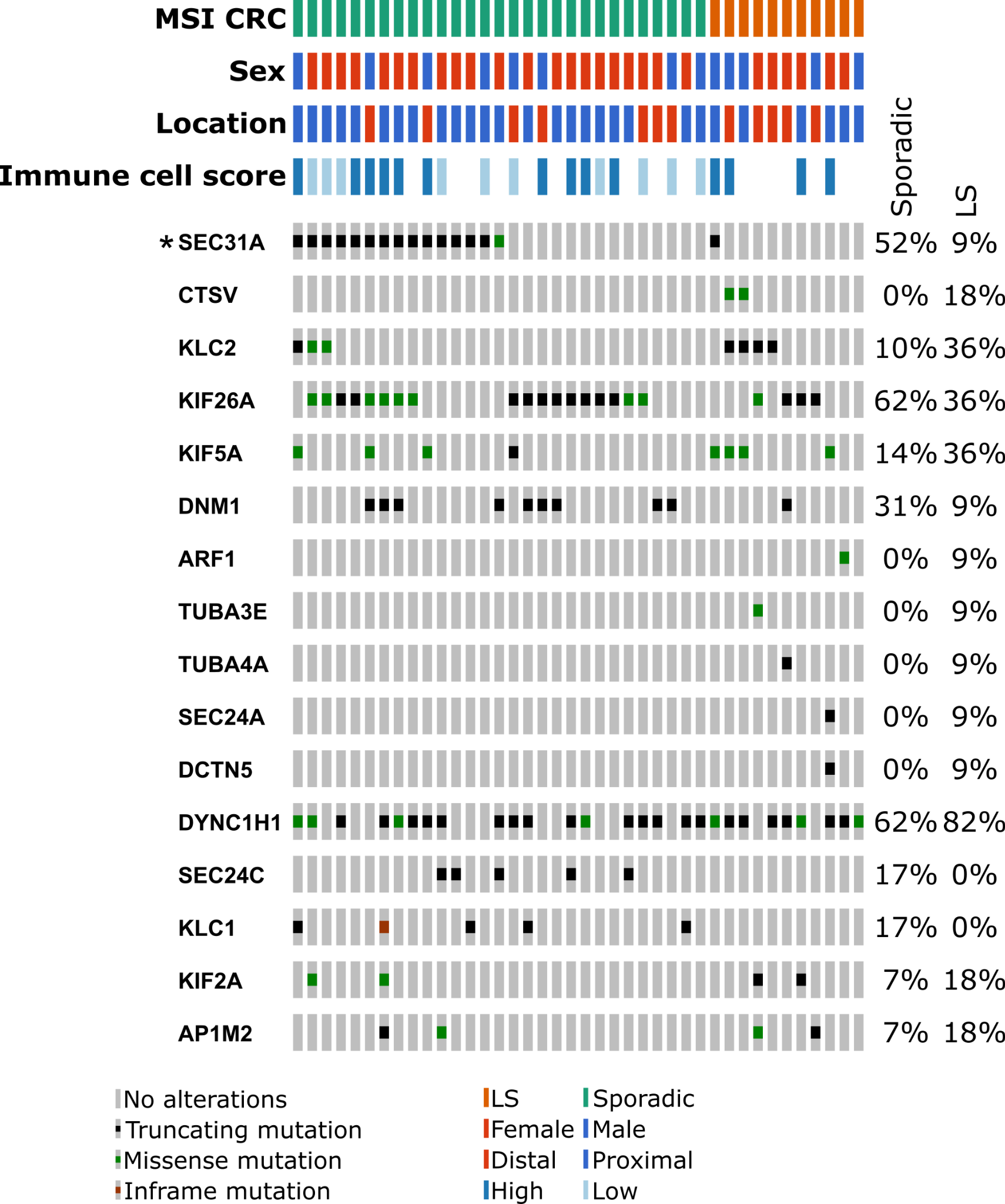


*Supplementary figure 16: Somatic non-synonymous mutations (SNVs and indels) in HLA class II antigen presentation-related genes in 29 sporadic MSI and 11 LS tumours. Genes are ranked by the differential variant frequency in sporadic MSI and LS indicated by Fisher's test P-values (* indicates the significant P-value, P=0.027). The top 16 genes are shown. Variant effect predictions required for the Oncoprint image were generated from BasePlayer somatic variant annotations* [*(Katainen et al. 2018)*](https://paperpile.com/c/2Mn114/W0UO)*. The percentage of samples carrying a mutation is indicated on the right-hand side.*

*
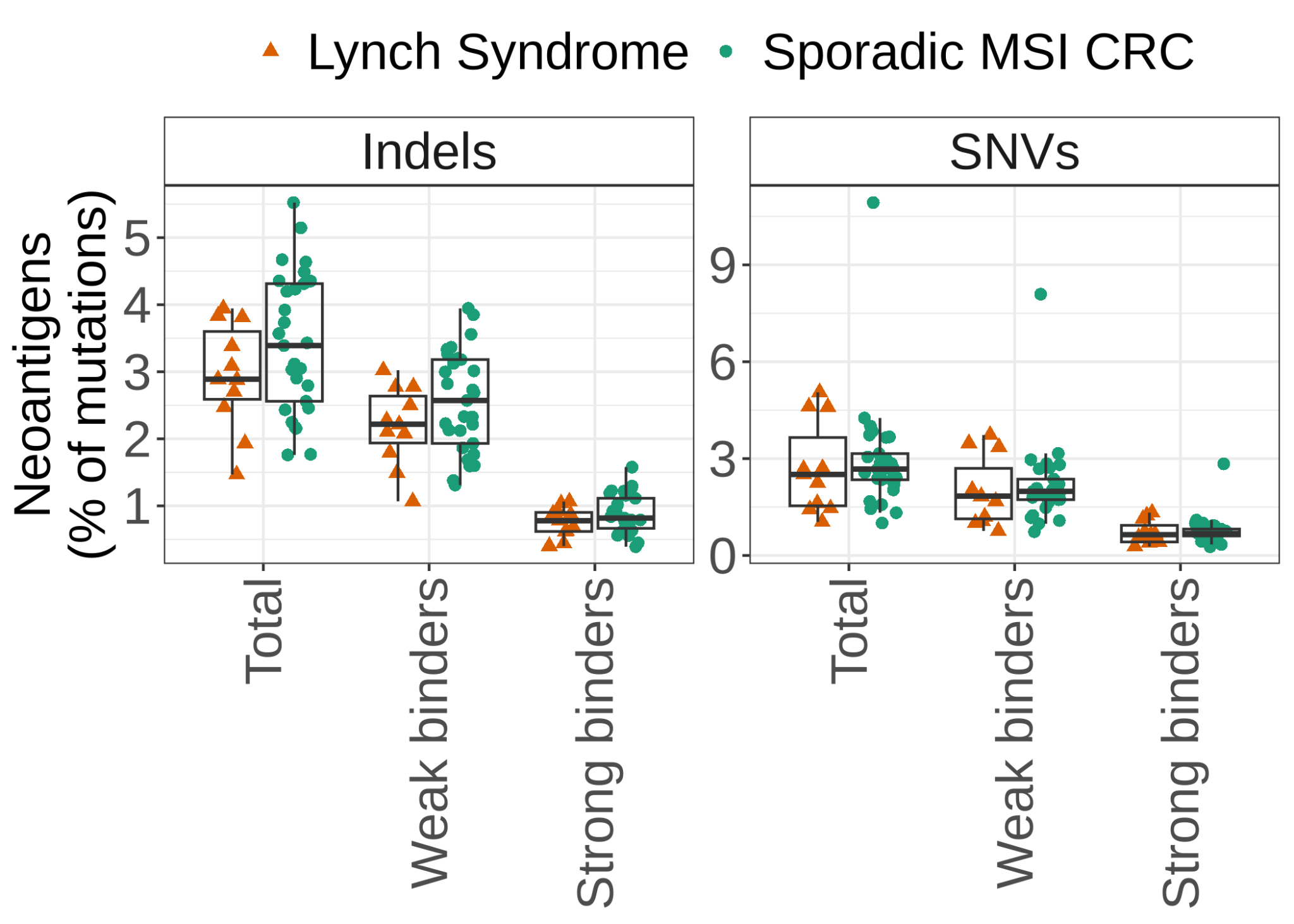
*

*Supplementary figure 17: The estimated total, weak binding and strong binding neoantigen counts normalised by the total number of indels and SNVs in each sample, in 11 LS and 29 sporadic MSI CRCs*

*
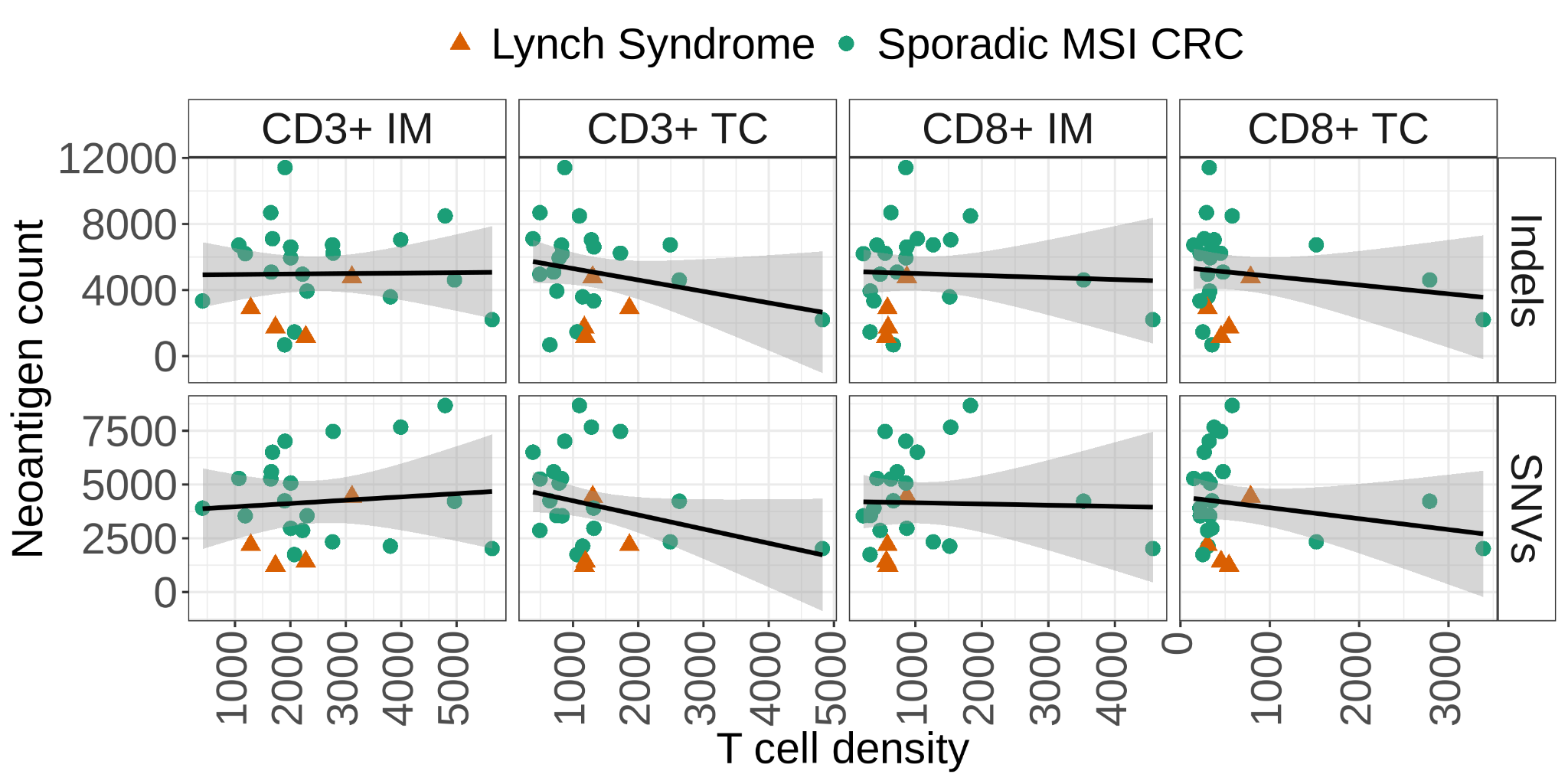
*

*Supplementary figure 18: The correlation between the total number of predicted neoantigens and the raw T cell densities.*

*
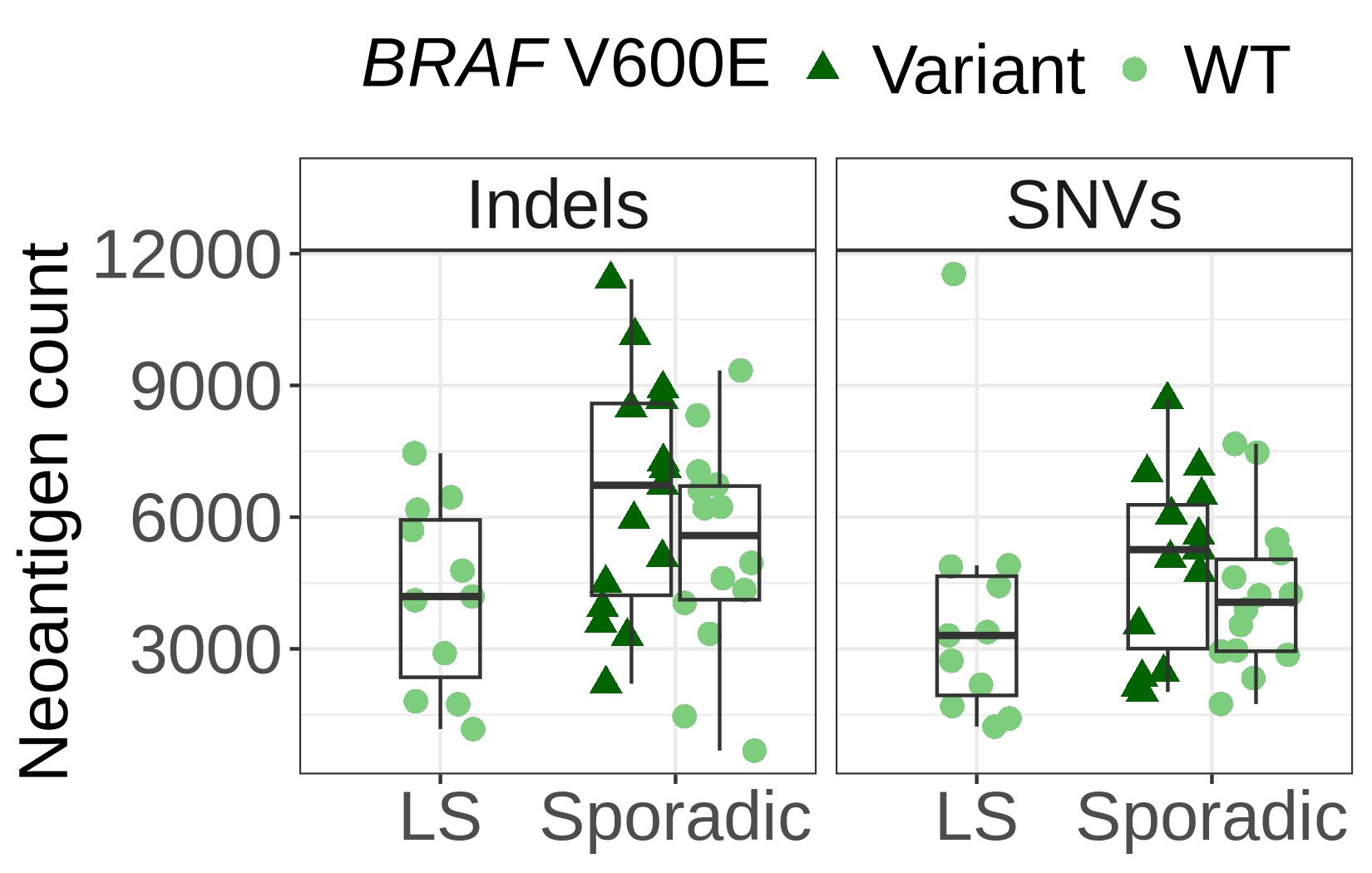
*

*Supplementary figure 19: The total number of predicted neoantigens in BRAF V600E mutant and wildtype (WT) tumours.*

*
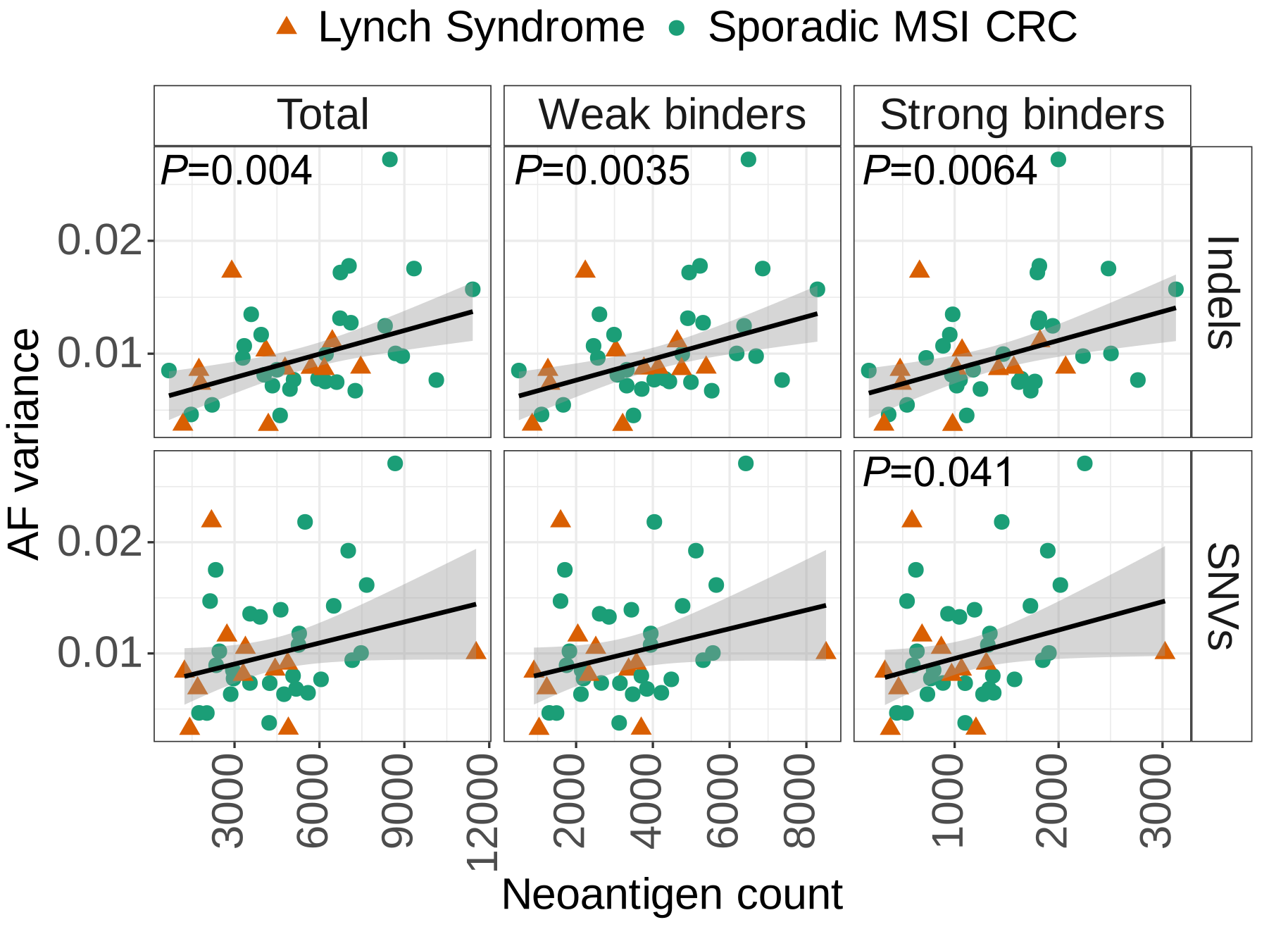
*

*Supplementary figure 20: The correlation between the predicted number of total, weak binding and strong binding neoantigens and the allelic fraction (AF) variance of indels and SNVs separately. Statistically significant correlations between AF variance and predicted neoantigen counts are indicated by P values (linear regression).*

## Supplementary Tables

| **Target** | **Clone** | **Host** | **Manufacturer** | **Catalog No.** | **Dilution*** | **Antigen retrieval **** |
| --- | --- | --- | --- | --- | --- | --- |
| CD66b | G10F5 | Mouse | Biolegend | 305102 | 02:40 | ER2, 20 min |
| CD16 | D1N9L | Rabbit | Cell Signaling | 24326S | 02:10 | ER1, 30 min |
| CD14 | D7A2T | Rabbit | Cell Signaling | 75181S | 02:10 | ER1, 60 min |
| HLA-DR | TAL 1B5 | Mouse | Santa Cruz | sc-53319 | 67:40:00 | ER1, 60 min |
| CD11b | D6X1N | Rabbit | Cell Signaling | 49420S | 02:00 | ER1, 30 min |
| CD68 | KP1 | Mouse | Biolegend | 916104 | 1:40 000 | ER1, 60 min |
| CD206 | E2L9N | Rabbit | Cell Signaling | 91992S | 09:20 | ER1, 30 min |
| CK | BS5 | Mouse | BioSite Histo | BSH-7124-1 | 07:40 | ER1, 60 min |

* BOND Primary Antibody Diluent (Leica Biosystems, AR9352) was used for antibody dilution.

** ER1, BOND Epitope Retrieval Solution 1: citrate based, pH 6.0 (Leica Biosystems, AR9961). ER2, BOND Epitope Retrieval Solution 2: EDTA based, pH 9.0 (Leica Biosystems, AR9640).

*Supplementary table 1: The antibodies used in the multiplex immunohistochemistry.*

| **Myeloid immune cell** | **Antibody marker combination** | **% of lineage (medians)** |
| --- | --- | --- |
| Tumour cells | CK+ |  |
| Total myeloid lineage cells | CD11b+ |  |
| Total granulocyte lineage cells | CD66b+ | 100 |
| Neutrophils | CD66b+ CD16+ | 89.5 |
| Eosinophils | CD66b+ Eosin+ | 1.1 |
| Immature granulocytic cells | CD66b+ CD16- | 10.5 |
| Total monocyte lineage cells | CD14+ | 100 |
| Mature monocytic cells | CD14+ HLADR+ | 93.5 |
| Immature monocytic cells | CD14+ HLADR- | 6.5 |
| CD16- monocytic cells | CD14+ CD16- | 21.8 |
| CD16+ monocytic cells | CD14+ CD16+ | 78.2 |
| Total macrophages | CD14+ CD68+ | 92 |
| M1-like macrophages | CD14+ CD68+ (HLADR/CD206 polarisation index; top 30%) | 25.1 |
| M2-like macrophages | CD14+ CD68+ (HLADR/CD206 polarisation index; bottom 30%) | 21.5 |

*Supplementary table 2: The Myeloid cell markers and the median percentage of the total lineage cells across all 27 MSI CRCs.*

| **Checkpoint Receptor** | **Protein name** | **Receptor - Alt Names** | **Checkpoint Ligand** | **Protein name** | **Ligand - Alt Names** |
| --- | --- | --- | --- | --- | --- |
| BTLA |  |  | TNFRSF14 |  | HVEM |
| TNFSF14 |  |  |  |  |  |
| CD27 |  | TNFRSF7 | CD70 |  | TNFSF7 |
| CD40 |  |  | CD40LG |  | CD40L |
| CD200R1 |  | CD200R | CD200 |  |  |
| CD244 |  | 2B4 | CD48 |  | SLAMF2 |
| CTLA4 |  | CTLA-4, CD152 | CD80 |  | B7-1 |
|  |  |  | CD86 |  | B7-2 |
| HAVCR2 |  | TIM-3 | LGALS9 |  | Galectin-9 |
| ICOS |  |  | ICOSLG |  | B7RP1, ICOSL, B7-H2 |
| PD1 |  | PD-1, CD279 | CD274 |  | PDL-1 |
|  |  |  | PDCD1LG2 |  | PDL-2, CD273 |
| PVRIG |  | CD112R | NECTIN2 |  | CD112 |
| TIGIT |  |  | PVR |  | CD155, Poliovirus receptor |
| TNFRSF4 |  | OX40 | TNFSF4 |  | OX40L |
| TNFRSF9 |  | 4-1BB, CD137 | TNFSF9 |  | 4-1BBL, CD137L |
| TNFRSF18 | GITR | CD357 | TNFSF18 | GITRL |  |
| TMIGD2 |  |  | HHLA2 |  | B7-H7 |
| ADORA2A |  | A2aR |  |  |  |
| CD28 |  |  |  |  |  |
| CD96 |  |  |  |  |  |
| CD226 |  | DNAM1 |  |  |  |
| ENTPD1 |  |  |  |  |  |
| LAG3 |  | LAG-3, CD223 |  |  |  |
| KIR3DL1 |  | KIR, CD158 |  |  |  |
| KLRC1 |  | NKG2A |  |  |  |
| NT5E |  | CD73 |  |  |  |
|  |  |  | BTN3A1 |  | CD277 |
|  |  |  | CD276 |  | B7-H3 |
|  |  |  | IDO1 |  | IDO |
|  |  |  | NCR3LG1 |  | B7-H6 |
|  |  |  | VSIR | VISTA |  |
|  |  |  | VTCN1 |  | B7x, B7-H4 |

*Supplementary table 3: The 48 immune checkpoint molecules. Receptor ligand pairing is not authorative.*
